# Design of tough adhesive from commodity thermoplastics through dynamic crosslinking

**DOI:** 10.1126/sciadv.abk2451

**Published:** 2021-10-15

**Authors:** Md Anisur Rahman, Christopher Bowland, Sirui Ge, Shree Ram Acharya, Sungjin Kim, Valentino R. Cooper, X. Chelsea Chen, Stephan Irle, Alexei P. Sokolov, Aditya Savara, Tomonori Saito

**Affiliations:** 1 Chemical Sciences Division, Oak Ridge National Laboratory, Oak Ridge, TN 37831, USA.; 2 Department of Materials Science and Engineering, University of Tennessee, Knoxville, TN 37996, USA.; 3 Materials Science and Technology Division, Oak Ridge National Laboratory, Oak Ridge, TN 37831, USA.; 4 Computational Sciences and Engineering Division, Oak Ridge National Laboratory, Oak Ridge, TN 37831, USA.; 5 Department of Chemistry, University of Tennessee, Knoxville, TN 37996, USA.

## Abstract

Tough adhesives provide resistance against high debonding forces, and these adhesives are difficult to design because of the simultaneous requirement of strength and ductility. Here, we report a design of tough reversible/recyclable adhesive materials enabled by incorporating dynamic covalent bonds of boronic ester into commodity triblock thermoplastic elastomers that reversibly bind with various fillers and substrates. The spectroscopic measurements and density functional theory calculations unveil versatile dynamic covalent binding of boronic ester with various hydroxy-terminated surfaces such as silica nanoparticles, aluminum, steel, and glass. The designed multiphase material exhibits exceptionally high adhesion strength and work of debonding with a rebonding capability, as well as outstanding mechanical, thermal, and chemical resistance properties. Bonding and debonding at the interfaces dictate hybrid material properties, and this revelation of tailored dynamic interactions with multiple interfaces will open up a new design of adhesives and hybrid materials.

## Teaser

Dynamic crosslinking of commodity thermoplastics with multiple interfaces enables remarkably tough reversible adhesion.

## INTRODUCTION

**Fig. 1.**
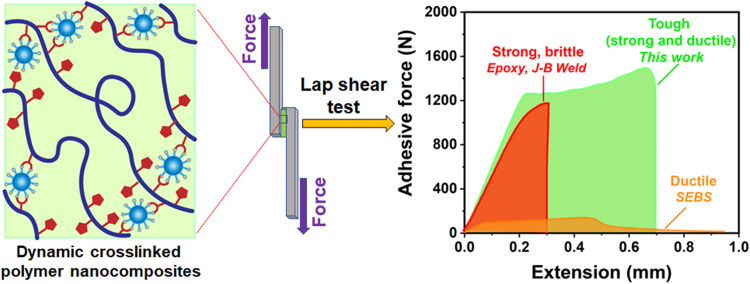
A schematic illustration of boronic ester dynamic covalent bond with the polymer matrix and SiNPs joining substrates with dynamically crosslinked nanocomposites (denoted as SiNP S-Bpin composites) and their lap shear adhesion test. The representative force-versus-extension curve for strong but brittle (commercial adhesives such as J-B Weld, red curve), ductile but weak (SEBS, orange curve), and tough (this work, green curve) adhesive. J-B Weld (epoxy) is one of the widely used strong adhesives, but its brittle nature results in low work of debonding (red curve); SEBS is widely used pressure-sensitive adhesive but very soft in nature (orange curve); SiNP S-Bpin composites exhibit very strong and tough adhesive behavior with very high work of debonding (green curve). Dynamic covalent B-O bonds between silica and polymer matrix make a stronger and tougher adhesive.

Adhesives have been used in all aspects of our daily life to connect materials temporarily or permanently (*1*). Synthetic polymers have been widely used as adhesive materials due to their capability to provide good contact between surfaces and dissipate energy under stress (*2*–*4*). Common adhesives are categorized into two types: strong adhesives or ductile adhesives. Load-bearing adhesives for structural applications including epoxies, polyurethanes, or acrylics typically provide strong adhesion, but their low work of debonding due to brittleness often leads to undesired cohesive failure (red curve in Fig. 1) (*5*). In contrast, ductile adhesives such as tape adhesives do not have strong adhesion but can dissipate mechanical stress through a soft matrix, preventing sudden bond failure (orange curve in Fig. 1) (*6*). Ductile adhesives are made of low modulus materials that limit their use in structural applications. The desirable tough adhesives having both characteristics of strong and ductile adhesion are extremely rare, because these combinations of adhesive property are difficult to attain due to their conflicting nature (*5*, *7*, *8*). Tough adhesives are characterized as having high degrees of debonding force (green curve in Fig. 1), which will provide improved safety and longevity of the structure with minimizing adhesive failures, and thus, successful development of remarkably tough adhesives will affect many applications in the electronic, construction, and automotive industries (*5*).

Nature has developed systems that exhibit both high strength and ductility (i.e., high toughness) in the same material, providing inspiration for the design of tough materials (*9*). For example, seashell nacre is one of the toughest natural materials, which consists of hexagonal platelets of aragonite linked together by elastic biopolymers (*10*, *11*). The hierarchical multiphase material design of soft-hard phases in nacre results in tough mechanical behavior by dissipating mechanical stress through the soft phase of the organic matrix. Marine mussel holdfast is another biological system that strongly binds to both organic and inorganic surfaces by assembling a crosslinked matrix of catechol-containing proteins (*12*, *13*). The catechol moieties in the mussel foot proteins use both covalent and noncovalent interactions with substrates via a combination of different bonding mechanisms adapted to different surfaces, resulting in remarkably versatile and strong adhesion (*14*–*18*). While many catechol-based adhesives are reported (*19*–*22*), these synthetic analogs are typically designed for wet surfaces, and they still tend to lack providing tough adhesion. By combining the inspiration of the mechanical toughness feature of nacre’s multiphase design and the dynamic bond adhesion feature of mussels, we sought to design a multiphase material incorporating interfacial dynamic bonding to develop extraordinarily strong and tough adhesives.

A tough adhesive needs to transmit force/energy between adhered substrates by having a stable mechanical hysteresis feature and form strong bonds with substrates (*6*, *23*). Typically, the addition of rubbery components into the brittle matrix can improve the toughness by dissipating energy through the rubbery segments (*24*). However, the occurrence of macrophase separation between elastomeric and brittle segments generally limits their formulations (*24*). A common approach to mitigate the issue of macrophase separation and brittleness of polymer is to use block copolymers such as a triblock copolymer having a rubbery midblock for extensibility and two glassy hard blocks for high modulus (*25*, *26*). Introduction of sacrificial hydrogen (H)–bonds into a polymer matrix can also enhance the adhesive toughness (*5*). There are a few reports on supramolecular adhesives incorporating H-bonding interaction that exhibit good adhesion on different surfaces (*27*–*32*). When adhesives are used for load-bearing application, they must have high cohesive molecular interactions and strong bonding ability with substrates (*33*). A conventional approach to prepare mechanically robust adhesives of high cohesion is the use of covalently crosslinked polymeric materials and the addition of fillers such as glass fiber, cellulose fiber (*19*), carbon nanotube, silica nanoparticles (SiNPs) (*3*, *34*), clay (*35*), graphene oxide (*36*), and cellulose nanocrystals (*23*) into the polymer matrix. However, most of the approaches involve irreversible covalent crosslinking, which are no longer able to reshape or rebond. These permanent adhesives are designed only for a single use, thus limiting their long-term stability (*37*). Permanent adhesives are also difficult to remove from the substrate and do not offer recyclability. There are a few reported studies on reversible adhesives by introducing covalent adaptable networks of disulfides (*23*, *38*–*41*), B-O bond (*42*), dynamic quaternization cross-links (*43*), transesterification reactions (*44*, *45*), and thiol-thioester exchange (*46*), while no reported studies extended dynamic covalent crosslinking to unmodified substrates for stronger interfacial bonding.

Here, we tailored the multiple interfacial interactions by incorporating dynamic chemistry into the adhesives. A dynamic covalent functionality of boronic ester was added to a commodity triblock thermoplastic elastomer, polystyrene-*b*-poly(ethylene-*co*-butylene)-*b*-polystyrene (SEBS), which allows dynamic covalent linkage with unmodified SiNPs. Boronic ester groups in the triblock copolymer react with hydroxyl groups on the SiNPs to yield dynamically crosslinked nanocomposites, which was further confirmed by density functional theory (DFT)–based calculations. The dynamic covalent bonds enable multiple reprocessings of these crosslinked composites and maintain their mechanical robustness. Thus, we hypothesized that the dynamic boronic ester groups on SEBS could also form covalent bonds with various oxide interfaces on the substrates for stronger adhesion. Furthermore, the soft ethylene butylene (EB) block in SEBS dissipates mechanical force, and the crosslinked nanocomposite structure provides mechanical robustness, while the boronic ester dynamic bonds enable the covalent bonding and rebonding ability with various substrates and SiNPs. This biomimetic multiphase composite produces a remarkably strong and tough adhesive (green curve in Fig. 1), revealing a simple and effective approach for the preparation of load-bearing tough adhesives. This versatile adhesive can be used in both dry and solution states and can provide strong and tough adhesion with various surfaces at both room and high temperature. These findings provide insights for another use of dynamic polymers for upcycling and will unwrap many opportunities for the design of exceptionally tough adhesives for many applications including automotive, aerospace, and construction industries.

## RESULTS

### Synthesis of multiphase material incorporating interfacial dynamic bonding

**Fig. 2.**
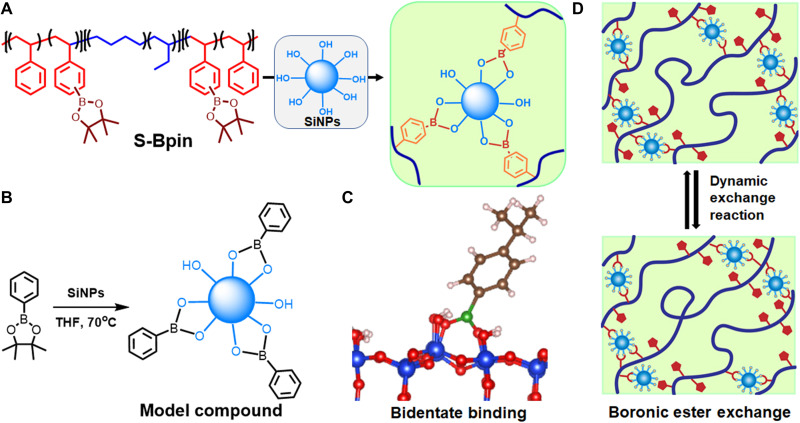
The design of a tough adhesive from commodity polymer. (**A**) Synthesis of crosslinked SiNP S-Bpin composites from S-Bpin and SiNPs, where S-Bpin was prepared from commodity polymer SEBS. (**B**) Synthesis of the model compound to confirm the formation of B-O bond from phenylboronic pinacol ester and SiNPs. (**C**) DFT calculations confirmed that covalent bonds can form between silicate surfaces and the polymer matrix (the bidentate binding geometry is depicted). (**D**) Dynamic boronic ester exchange crosslinking reaction between hydroxyl groups on SiNPs and boronic ester groups of S-Bpin affords reprocessable materials.

SEBS triblock copolymer (118 kg/mol) with 30 mole percent (mol %) styrene was modified via aromatic C-H borylation to incorporate dynamic boronic ester functional groups to yield a borylated SEBS triblock copolymer (S-Bpin) (Fig. 2A) (*47*). The successful S-Bpin conversion was evidenced in the ^1^H nuclear magnetic resonance (NMR) (fig. S1) and Fourier transform infrared spectroscopy (FTIR) spectra (fig. S2). The aromatic C-H protons of styrene in the ^1^H NMR spectrum split into three broad peaks at the 6.0- to 7.8-ppm (parts per million) region, indicating successful functionalization on the aromatics, while pinacol boronate ester (Bpin) methyl protons were overlapped with the SEBS backbone methylene peak. The degree of aromatic ring functionalization was calculated from ^1^H NMR spectrum of S-Bpin and SEBS (fig. S1) based on the relative intensity of the methyl group in 1,2-butylene unit of the polymer chain (at 0.78 to 0.88 ppm) with respect to the increased integral ratio of the overlapping SEBS-methylene and Bpin methyl resonance (at 0.9 to 1.5 ppm). ^1^H NMR spectrum indicates that a total of 95 mol % of aromatic rings on the styrene block are functionalized by Bpin. The presence of clear signals at 1350 and 1123 cm^−1^ for asymmetric and symmetric stretching of the B-O bond in the FTIR spectrum (fig. S2) further confirmed the successful borylation. Similarly, the polystyrene homopolymer was also modified via aromatic C-H borylation to elucidate the impact of triblock architecture on the adhesive properties (fig. S3).

The biomimetic multiphase composites of S-Bpin with SiNPs (denoted as SiNP S-Bpin composites) were prepared with varying amounts of SiNPs (size of ~14 nm) and S-Bpin, where SiNPs acted as a dynamic crosslinker (Fig. 2A). The tetrahydrofuran (THF) solution of S-Bpin and different weight percent (wt %) of SiNPs were mixed at room temperature and dried under reduced pressure at 120°C to obtain partially crosslinked networks. Fully cured SiNP S-Bpin composite films were subsequently prepared by hot pressing at 215°C for 3 hours under ~0.38-MPa pressure. The crosslinking reaction (chemical reaction) between hydroxyl groups on SiNPs and boron pinacol ester groups of S-Bpin was confirmed by FTIR spectrum, where a broad signal appeared at 1114 cm^−1^ corresponding to the Si-O bond overlapped with a B-O bond (fig. S2). The obtained clear S-Bpin solution in THF with SiNP loading of 5 to 20 wt % (fig. S4A) indicates good miscibility of SiNPs and S-Bpin polymer. In contrast, SiNPs added to the SEBS solution in THF at any loading exhibited a cloudy solution (fig. S4B). Because of the interaction between SiNPs and polymer matrix, up to 20 wt % SiNP loading maintained high dispersibility. The high dispersion of SiNPs is also confirmed by the transmission electron microscopy (TEM) images (fig. S5, A and B). The SiNP S-Bpin composite solution with SiNP loading above 20 wt % formed a cloudy solution, which implies SiNP aggregation or macrophase separation between excess silica and polymer matrix. The microphase separation was maintained for the crosslinked SiNP S-Bpin composites, evidenced by the presence of a strong primary scattering peak (d-spacing of ~30 nm) in small-angle x-ray scattering (SAXS) profile of 10 wt % SiNP S-Bpin (fig. S5C). To confirm the reaction between SiNPs and the boronic ester group of S-Bpin polymer, one small molecular model compound was synthesized from phenylboronic acid pinacol ester and SiNPs following a conventional silica modification reaction condition (*48*). Phenylboronic acid pinacol ester was treated with SiNPs in THF at 70°C to form SiNPs modified with phenylboronic acid as shown in Fig. 2B. ^1^H NMR spectrum (fig. S6) shows the disappearance of the peak at 1.3 ppm for four methyl groups in pinacol from the phenylboronic acid pinacol ester, indicating successful conversion. The weight gain on the SiNP surface in the thermogravimetric analysis (TGA) curve (fig. S7) also confirmed the boronic ester transesterification reaction.

To further understand the feasibility and the energetics of the reaction between hydroxyl groups on SiNPs and the boronic ester groups on S-Bpin, we performed DFT calculations. DFT calculations indicate that covalent bonding can occur between the boron ester moieties and the hydroxyl groups on the silica surface via either a monodentate fashion or a bidentate fashion (Fig. 2C and fig. S17). During this binding, one or more Si-O-H moieties are converted to Si-O-B, where the monodentate bindings are lower energy states than the bidentate bindings. The monodentate binding energy of boron ester with silica termination was estimated to be ~70 kJ/mol. The binding energy is significantly higher than typical secondary interactions such as hydrogen bonding (4 to 13 kJ/mol) but lower than the covalent C-C bond (~356 kJ/mol) (*49*). The high binding energy of boron ester with silica surfaces suggests that S-Bpin crosslinking by SiNPs can behave similarly to covalently crosslinked composite network, with the capability of this B-O bond exchange with neighboring hydroxyl groups on SiNPs, which enables the reprocessability (Fig. 2D).

The crosslinking nature of SiNP S-Bpin composites was demonstrated via a solvent resistance study. As shown in fig. S8A, composite films were subjected to different solvents such as dichloromethane (DCM), chloroform (CHCl_3_), THF, dimethylformamide (DMF), and deionized water for 7 days at room temperature, and the solubility was monitored. The crosslinked samples of SiNP S-Bpin composites were not dissolved in any solvents, although some degree of swelling was observed in DCM, CHCl_3_, and THF. In contrast, uncrosslinked S-Bpin was readily dissolved in THF within a few minutes. It should be noted that the S-Bpin also exhibited high hydrolytic stability as it absorbed negligible water around 2.2 wt % after 7 days of immersion in water. There is no significant change observed in the TGA curve (fig. S8B) and FTIR spectrum (fig. S8C) that confirmed the stability of boronic ester bonds with the presence of water due to the embedded nature of boronic ester groups by local hydrophobic polymer chains, which is also observed by others (*50*, *51*).

### Mechanical properties of dynamic crosslinked multiphase composites

**Fig. 3.**
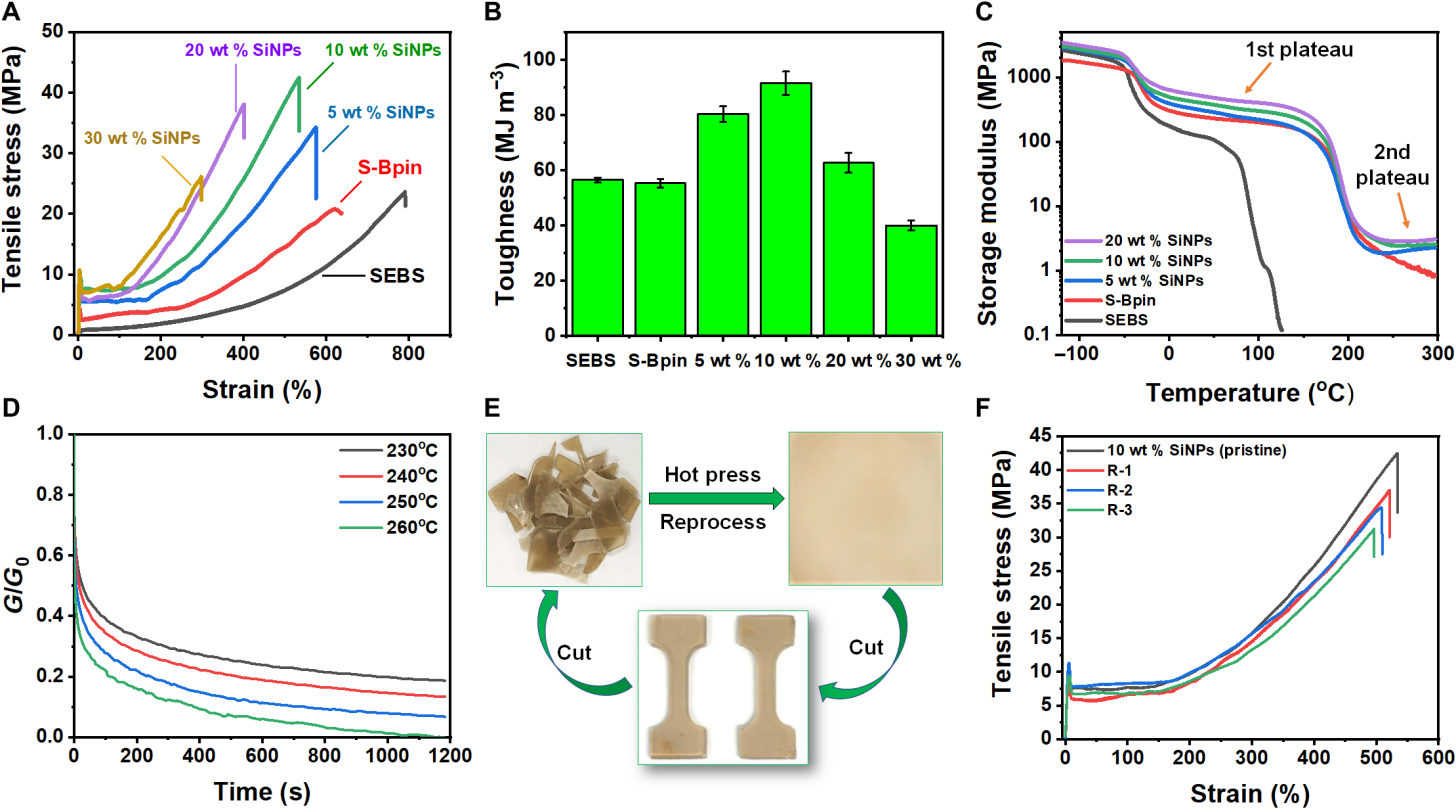
Mechanical properties of SEBS, S-Bpin, and different SiNP S-Bpin composites. (**A**) Tensile stress-strain curves measured by tensile test. (**B**) Toughness measured from the area underneath of tensile stress-strain curves. (**C**) Shear storage modulus as a function of temperature measured by DMA. (**D**) Stress relaxation of 10 wt % SiNP S-Bpin composites at a temperature range of 230° to 260°C. (**E**) Schematic representation of polymer reprocessability. Photo credit: Md Anisur Rahman, ORNL. (**F**) Tensile stress and strain properties of reprocessed 10 wt % SiNP–loaded samples. All error bars represent the SD with at least three replicates.

The mechanical properties of SEBS were significantly improved after modification and subsequent crosslinking by SiNPs, as observed in the stress-strain curves (Fig. 3A). The presence of a clear yield point in tensile stress-strain curves of SiNP S-Bpin nanocomposites indicates the elastic-to-plastic transition with subsequent strain hardening. Compared to SEBS copolymers, the crosslinked nanocomposites exhibited significantly higher tensile strength and Young’s modulus with slightly decreased elongation at break. The SiNPs act not only as a reinforcer but also as dynamic covalent crosslinkers through B-O bond formation between the silicate surfaces and polymer matrix. The higher loading of SiNPs increases the cross-link density that enhances the mechanical strength, while the restriction to the polymer chain mobility results in the decrease of elongation at break. The tensile strength and toughness of 10 wt % SiNP S-Bpin achieved 40 MPa and 91.5 MJm^−3^, respectively (table S1), which are almost double those of SEBS (25 MPa and 56.5 MJm^−3^). The tensile strength and toughness of 20 wt % SiNP S-Bpin resulted in 32 MPa and 62.6 MJm^−3^, whereas 30 wt % exhibited 26 MPa and 40.01 MJm^−3^ (Fig. 3B). The mechanical properties decreased above 20 wt % SiNP loading probably due to the aggregation of unreacted SiNPs. The Young’s modulus also increases significantly with SiNP loading (table S1). For example, Young’s modulus of SEBS increases from 14.5 MPa to 368 MPa with 20 wt % SiNP–loaded composites.

The boronic ester–based crosslinking significantly broadens the service window of SEBS, which is important for many applications with a required wide operation temperature range. The dynamic mechanical analysis (DMA) data (Fig. 3C and fig. S9) indicate a significant shift in the glass transition temperature (*T*_g_) of polystyrene block from ~90°C in SEBS to ~200°C in the modified SEBS (table S1), while the minimal change is observed in *T*_g_ for EB block (from −40°C to −30°C). The *T*_g_ of crosslinked SiNP S-Bpin composites exhibits an additional slight increase with increasing SiNP loading, probably due to the formation of higher crosslinked networks that retards polystyrene chain mobility. For example, the *T*_g_ of the polystyrene block in the 20 wt % SiNP–loaded composite is 211°C compared to *T*_g_ of ~204°C for the 5 wt % SiNP S-Bpin. At the rubbery plateau range of −30° to 200°C, the storage modulus also increases with SiNP loading. As shown in Fig. 3C, the 20 wt % SiNP S-Bpin composite shows higher storage modulus (553 MPa) than SEBS (126 MPa) and S-Bpin (221 MPa) at room temperature. The DMA curves for SiNP S-Bpin samples (Fig. 3C) also show the second plateau region after the second *T*_g_, indicating the formation of strong dynamic covalent crosslinking between silica and polymer matrix.

The dynamic behavior of boronic ester exchange in crosslinked 10 wt % SiNP S-Bpin (Fig. 3D) and 20 wt % SiNP S-Bpin (fig. S10A) composites is demonstrated in the stress relaxation experiment by monitoring stress decay at elevated temperature (230° to 260°C) over time at a constant strain of 2%. The crosslinked SiNP S-Bpin composites show substantial stress decay with time, and their stress relaxation rate is increased with the temperature above *T*_g_ (Fig. 3D and fig. S10A) because the boronic ester transesterification dynamic exchange is accelerated at a higher temperature (*52*). The composites with lower SiNPs exhibited much faster relaxation when compared with higher SiNP-loaded samples (fig. S10B) because the higher SiNP content increases the cross-link density and retards the topology rearrangement (*53*, *54*). The characteristic relaxation times (τ) of 10 and 20 wt % SiNP S-Bpin were determined at 1/*e* (37%) of the normalized relaxation modulus (table S2). The relaxation time (τ) of 10 wt % SiNP S-Bpin at 250°C (35 s) is more than eight times faster than that of 20 wt % SiNP S-Bpin (300 s), suggesting that the higher SiNPs form a higher degree of crosslinked network, which results in the restricted chain mobility and hindered bond reshuffling. The apparent activation energies (*E*_a_) for stress relaxation of 10 and 20 wt % SiNP S-Bpin are in the range of 150 to 170 kJ/mol, obtained from fitted curves of the relaxation time versus temperature plot (fig. S10C). This apparent activation energy is relatively higher than those reported in previous studies (*52*–*54*), which may be due to densely crosslinked microphase separated domain from high–molecular weight block copolymer matrix and restricted diffusion of reactive associative functional groups.

The boronic ester crosslinked nanocomposite samples can be easily reprocessed at a high temperature as the B-O bond can break and reform or by rearranging the network at above *T*_g_. Because the *T*_g_ of the polymer (outer block) is around 200° to 212°C, a high temperature above *T*_g_ around 215°C is required to allow polymer chain mobility and network adaptability for reprocessing. The composite film was cut into small pieces and reprocessed at 215°C and ~0.38-MPa pressure for 2 hours (Fig. 3E). The efficacy of reprocessability was evaluated by measuring mechanical properties of reprocessed samples including tensile stress and strain, as shown in Fig. 3F. The reprocessed samples showed slightly diminished tensile strength and elongation at breaks after the third cycle (fig. S11A), which may be due to the thermal oxidation as the samples were reprocessed at high temperatures in an open-air condition. The TGA curves showed their high stability against thermal degradation at elevated temperatures (fig. S11B), and their chemical composition did not change significantly upon reprocessing as evidenced by the FTIR spectra (fig. S11C). Performing three reprocessing cycles of the crosslinked nanocomposite samples corroborates the dynamic nature of the B-O bond exchange between the silicate surfaces and polymer matrix. These SiNP S-Bpin composites can be used as reprocessable crosslinked polymeric materials and can serve as next-generation sustainable hybrid materials such as reusable adhesives and parts.

### Tough adhesive behavior

As S-Bpin can form dynamic covalent bonds with hydroxyl groups on SiNPs, we hypothesized that S-Bpin would show strong adhesion with hydroxyl-terminated surfaces. DFT calculations (see details in the Supplementary Materials) were used to explore covalent bonding between the boron ester moieties of S-Bpin and the hydroxyl-terminated aluminum, steel, and glass surfaces. In general, glass surfaces are terminated with Si-O-H groups, whereas aluminum and steel metal terminate in an oxidized layer of Al-O-H and Fe-O-H, respectively (*31*, *55*–*57*). DFT calculations indicate that covalent bonding can form with hydroxyl groups found on all of these surfaces via either a monodentate or a bidentate fashion, where the bidentate states are less energetically favored and require higher temperatures to form the bidentated geometry. The monodentate bindings of S-Bpin with Si-O-H, Al-O-H, and Fe-O-H were calculated to have binding energies of 72, 16, and 17 kJ/mol. The ultimate binding might include a mixture of monodentate bindings and bidentate bindings but could be dominated by either type of bonding. According to the DFT results, the S-Bpin polymer should show strong adhesive behavior with hydroxy-terminated surfaces, and the higher adhesion on the glass can be rationalized either by stronger binding energy or due to access to a larger number of hydroxyls. To investigate the adhesive property of S-Bpin experimentally, the lap shear adhesion test was performed on aluminum (Al) surfaces initially with an overlapped surface area of (12 mm × 12 mm) 144 mm^2^ following a modified version of ASTM D1002. S-Bpin exhibited a lap shear strength of 4 MPa, while the lap shear strength of SEBS on an Al substrate resulted in 2.5 MPa. SEBS is widely used as a hot-melt pressure-sensitive adhesive, and the improved adhesion of S-Bpin indicates that S-Bpin has a stronger interaction with Al compared to SEBS as predicted by DFT calculations. Although the bonding strength was improved for S-Bpin on Al, the cohesive failure was observed on the joint. The addition of SiNPs mitigates the issue and improves the cohesive force of S-Bpin by the combination of physical interactions (e.g., hydrogen bonding or van der Waals interactions) and dynamic covalent crosslinking.

**Fig. 4.**
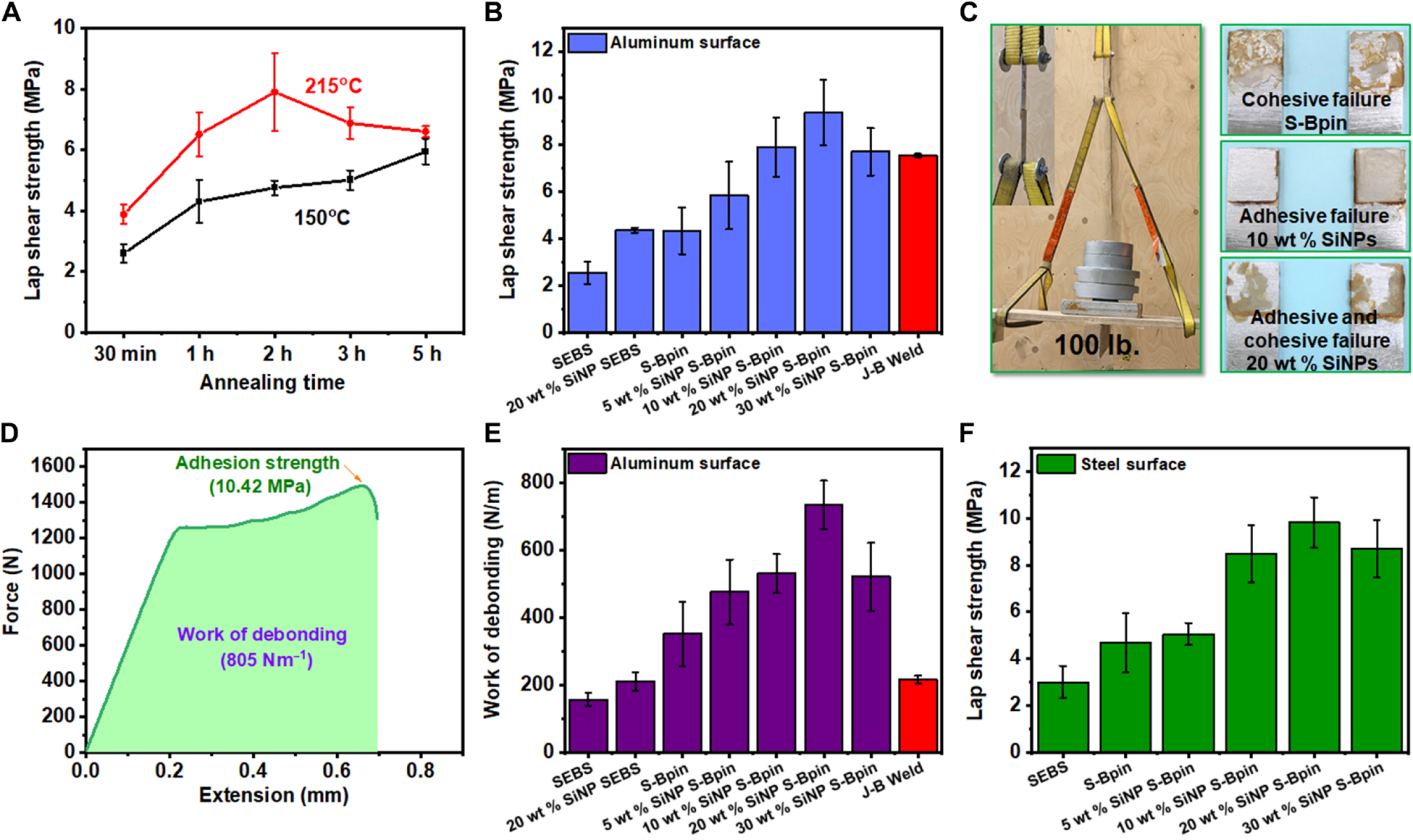
Lap shear adhesion tests. (**A**) Annealing time and temperature effect on the lap shear strength study using aluminum substrates. (**B**) Effect of SiNP loading on lap shear strength on aluminum substrates. (**C**) Load-bearing ability and inset image showing the adhesive joint of two aluminum plates. Photo credit: Christopher Bowland, ORNL. Cohesive and adhesive failure of lap joint after adhesion test. (**D**) Force-versus-extension curve for 20 wt % SiNP composite to show work of debonding and lap shear adhesion strength on aluminum substrates. (**E**) Work of debonding for SEBS, S-Bpin, different silica-loaded nanocomposites, and J-B Weld (commercial epoxy-based glue) on aluminum substrates. (**F**) Effect of SiNP loading on lap shear strength on steel substrates. All error bars represent the SD with at least three replicates.

The adhesion of 10 wt % SiNP S-Bpin composite was initially investigated using an Al surface to understand the effect of curing time, processing temperature, and amount of adhesive. The SiNP S-Bpin composite in THF solution with a concentration of 100 mg/ml was directly placed on top of the Al surface; then, another Al surface was put on top, held together for 1 min, and dried under vacuum at 120°C for 4 hours (fig. S12A); and subsequently, the lap shear strength was measured (fig. S12B). The adhesion strength of the as-prepared 10 wt % SiNP S-Bpin was around 2.5 MPa. At this curing condition, SiNP S-Bpin composites are not fully cured, which does not provide efficient boron ester bonding with hydroxyls on the substrate and rather allows polymer chains to move and slip easily. To explore the optimum curing time and temperature, a 10 wt % SiNP S-Bpin composite was tested against two temperatures, 150°C (below *T*_g_) and 215°C (above *T*_g_), with different curing times under ~0.096-MPa contact pressure (Fig. 4A). With 150°C curing temperature, the lap shear strength increased with a longer curing time. In contrast, the lap shear strength reached a maximum adhesion value after 2-hour curing at 215°C and decreased beyond 2-hour curing. At 215°C, polymer chains rearrange and reach optimum curing at 2 hours to provide stronger adhesion by activating chemical (dynamic B-O bond) and physical interactions (hydrogen bonding or van der Waals interaction) that enhance dynamic network adaptability and better surface wettability. The temperature above *T*_g_ also allows the dynamic bonds to exchange and creates better adhesive layers with hydroxy-terminated surfaces. Curing more than 2 hours might result in over-crosslinking or partial oxidation (as observed from slight color change) that may cause the material to become brittle and decrease adhesion strength. We also investigated the minimum amount of composite solution required for better adhesive properties. The maximum adhesion was obtained for 200 to 300 μl of the composite solution of a concentration of 100 mg/ml for the 10 wt % SiNP S-Bpin composite (fig. S12C). Thus, all the subsequent adhesion tests were performed using 200 μl of solution (100 mg/ml) onto 144 mm^2^ area and cured for 2 hours at 215°C.

The lap shear adhesion on Al surfaces of SiNP S-Bpin composites with different SiNP loading corresponds well to the balance of ductility and strength, namely, the adhesive property correlates well with enhanced mechanical properties (e.g., modulus). The lap shear strength increased from 4 to 10.4 MPa with increasing SiNP loading from 0 to 20 wt % but decreased above 20 wt % SiNP loading, indicating the optimum cross-link density to attain the strong-ductile adhesive property of the polymer (Fig. 4B). Increasing the amount of SiNPs improves mechanical modulus that enhances the overall mechanical strength and cohesive force of the materials, as observed in the strong cohesiveness of 10 and 20 wt % samples resulting in an adhesive failure (Fig. 4C). Above 20% SiNP loading, the decreased ductility and cohesive force lead to a decrease in adhesive properties. Modulus at the operating condition plays an important role in the lap shear strength of an adhesive (*23*, *58*). Higher modulus at operating conditions generally contributes to increasing lap shear strength. For example, the lap shear strength increases from 5.8 MPa in 5 wt % SiNP S-Bpin to 7.9 MPa in 10 wt % SiNP S-Bpin, with the corresponding increase of Young’s modulus from 249.5 to 288 MPa. In addition, 20 wt % sample showed very high storage modulus (553 MPa) and Young’s modulus (368 MPa) at room temperature and low modulus at high temperatures (above 215°C) that favor stronger adhesive bonding. The 20 wt % SiNP S-Bpin sample not only shows higher mechanical modulus but also provides the optimum balance of adhesive and cohesive forces, where simultaneous adhesive and cohesive failure, as shown in Fig. 4C, typically corresponds to the highest adhesive strength (*13*). The lap shear adhesion of 20 wt % SiNP SEBS was also measured and compared with 20 wt % SiNP S-Bpin to investigate the relative contribution of the surface chemical bonding. The lap shear adhesion of 20 wt % SiNP S-Bpin is approximately three times higher than that of 20 wt % SiNP SEBS (Fig. 4B), indicating the strong contribution by the formation of dynamic B-O bond with hydroxy-terminated surface.

The outstanding toughness of these SiNP S-Bpin composite adhesives are observed in force-extension curves (fig. S12D), where the curves for S-Bpin and all SiNP S-Bpins exhibit a sharp rise followed by a gradual increase in the force until failure, suggesting the presence of ductile, plastic behavior. To the best of our knowledge, this type of ductile adhesive behavior is very rare for any commercial adhesives and any literature reports on adhesives. The integrated area underneath the force-extension curve is defined as the work of debonding, or work of adhesion, namely, energy required to break the adhesive joint (Fig. 4D) (*5*). We calculated the work of debonding for all the SiNP S-Bpin composites and compared them with SEBS, 20 wt % SiNP SEBS, and S-Bpin (Fig. 4E). The work of debonding for the 20 wt % SiNP S-Bpin is 733.96 ± 71.58 Nm^−1^, approximately five times higher than that of SEBS (157.25 Nm^−1^) and more than three times higher than that of 20 wt % SiNP SEBS (211 Nm^−1^) and that of commercial J-B Weld epoxy-based glue (226.4 Nm^−1^) (Fig. 4E). Most of the existing commercial adhesives are brittle, giving them lower values on the work of debonding. The incorporation of SiNPs into S-Bpin provides the increase in the modulus without losing extensibility, the work of debonding, and the overall toughness of the adhesives. The enhancement of toughness, adhesion strength, and work of debonding was achieved only after the modification of SEBS and introduction of SiNPs into the S-Bpin, as they can form a strong dynamic B-O covalent bond with hydroxyl-terminated surfaces. Furthermore, the EB blocks of triblock copolymer distribute mechanical stresses to prevent sudden breakage. In contrast, boronic ester functionalized polystyrene homopolymer is too brittle, and thus, the adhesion could not be measured, which also indicates the importance of triblock copolymer architecture for leading to tough adhesives. These tough SiNP S-Bpin composite adhesives can be applied in structural applications, as they can dissipate mechanical stress throughout the joints and prevent abrupt failure. These tough adhesives will provide long-lasting high load-bearing capability and will prevent premature adhesive failures in many applications.

**Fig. 5.**
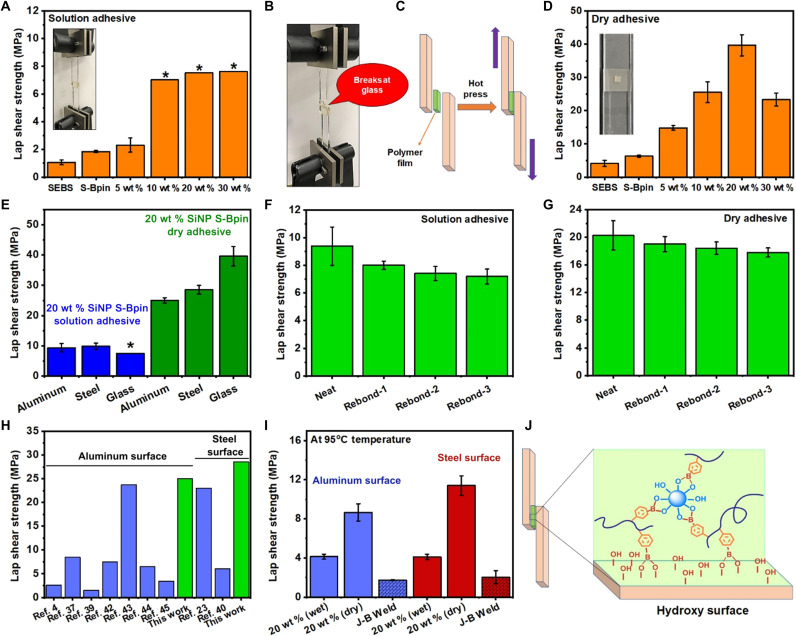
Lap shear adhesion on glass substrate, adhesive reusability, and comparison to the other adhesives. (**A**) Lap shear adhesion on glass surfaces using the composite solution. * indicates that the adhesion could go a much higher value, but we were unable to measure because of glass substrate failure. (**B**) Lap shear adhesion setup for glass showing the glass fracture rather than breaking the adhesive bonds. (**C**) Graphical representation of lap shear adhesion test for composite films. (**D**) Lap shear adhesion on a glass surface using composite films with reduced adhesive cross-section area of (3 mm × 3 mm) 9 mm^2^ and inset image showing the lap shear adhesion setup. (**E**) Comparison of adhesive performance of 20 wt % SiNP S-Bpin sample on different substrates. (**F**) Rebonding ability tests for the 20 wt % SiNP S-Bpin solution on Al surface with adhesive cross-section area of (12 mm × 12 mm) 144 mm^2^. (**G**) Rebonding ability tests for the 20 wt % SiNP S-Bpin composite film on Al surface with adhesive cross-section area of (6 mm × 6 mm) 36 mm^2^. (**H**) Comparison of lap shear adhesion of dynamic covalent bond–based adhesives reported in literatures (relevant data and references are summarized in table S3). (**I**) Adhesive performance of 20 wt % SiNP S-Bpin composite solution and dry film on Al and steel surfaces at 95°C. (**J**) Proposed mechanism of adhesion procedure. Photo credit: Md Anisur Rahman, ORNL. All error bars represent the SD with at least three replicates.

SiNP S-Bpin composites exhibit versatile adhesion to various surfaces. The lap shear adhesion test results on a steel surface showed a similar trend to that of the Al surface (Fig. 4F). As shown in the force-versus-extension curve in fig. S13A, 20 wt % SiNP S-Bpin composite exhibited tough adhesive performance on a steel surface, where the lap shear adhesion and work of debonding (fig. S13B) reached 10 MPa and 1103 Nm^−1^, respectively. In the case of the glass surface, the lap shear adhesion test was first performed with (14 mm × 6 mm) 84 mm^2^ adhesive joints using 50 μl of composite solution after curing at 215°C for 2 hours. The lap shear results (Fig. 5A) show that S-Bpin and SiNP S-Bpin composite samples have very strong adhesion on the glass surface, which is consistent with the strong binding calculated by DFT. The nanocomposites with SiNP loading above 5 wt % exhibited too strong adhesive force that resulted in the glass substrate failure every time, while adhesive bonds remained intact (Fig. 5B). To solve this issue, a smaller area of adhesive was used, which reduced the force required to break the adhesive joints. Recently, Rowan and coworkers used an adhesive polymer film to test the adhesion on a very small area on glass substrates (*23*), and we adopted the method because controlling a smaller adhesive area is difficult for adhesive solutions. We measured the lap shear adhesion using (6 mm × 6 mm) 36 mm^2^ and (3 mm × 3 mm) 9 mm^2^ composite films with a constant film thickness of around 25 to 30 μm. The SiNP S-Bpin composite film was placed between two glass sheets (Fig. 5C) and hot-pressed at 215°C for 2 hours with constant pressure (1.38 MPa) and observed that 9 mm^2^ bonded samples showed adhesive failure, while 36 mm^2^ bonded area still resulted in glass side break (structural failure). SiNP S-Bpin composites exhibited remarkably strong bonding on the glass substrates, in which an increasing adhesion strength was achieved with an increased amount of SiNP loading. Similar to the Al and steel surfaces, the maximum lap shear adhesion was obtained for the 20 wt % SiNP sample, resulting in unprecedented 39.6 ± 3.2 MPa (Fig. 5D). To compare the adhesion strength of glass with that of metals, the adhesive behavior of 20 wt % SiNP S-Bpin composite dry adhesive film with a smaller area of 9 mm^2^ was measured for Al and steel surfaces by the identical procedure. The lap shear strength on Al and steel surfaces exhibits 25.01 and 28.54 MPa, respectively, more than 10 MPa lower than the adhesion on the glass surface (39.6 MPa) (Fig. 5E). It should be noted that the lap shear adhesive strength of the different adhesive surface areas of 36 and 9 mm^2^ shows an insignificant difference (fig. S14) (*23*). The exceptionally strong adhesion on glass is due to the presence of hydroxyl groups that give rise to covalent bonds with the boron ester and potentially the formation of additional hydrogen bonding between hydroxyl groups of the polymer nanocomposite and the glass surface. The glass surface has a higher density of hydroxyl groups per given area, leading to stronger adhesion. In addition, the surface energies for glass are higher than metals, which may lead to enhanced wetting and higher adhesion strength (*23*).

Conventional structural adhesives are single-use adhesives with irreversible adhesion, which also leave strong adhesive residues when detached. Here, SiNP S-Bpin composite-based adhesives can be rejoined even after complete detachment due to the presence of boronic ester–based dynamic covalent bonds. The dynamic B-O bond allows rebonding and helps to dissipate energy or mechanical stresses. This is an important aspect of the developed adhesives because traditional adhesives such as epoxy and cyanoacrylate-based super glue are not capable of rebonding as they are made of permanently crosslinked materials. The rebonding capability was investigated by a repetitive sequence of breaking the lap joint at room temperature and rebonding to the Al surface at 215°C. As shown in Fig. 5 (F and G), the 20 wt % SiNP S-Bpin composite sample (solution and dry) showed favorable retention of lap shear adhesion even after the third rebonding cycle.

To evaluate the efficacy of our biomimetic multiphase composite-based adhesive for practical applications, we compared their adhesion strength with widely used commercial glues (Table 1) and recently reported dynamic polymer-based adhesives (Fig. 5H and table S3). In addition to a typical melt adhesive SEBS, four different types of representative adhesives including Loctite super glue (ethyl cyanoacrylate), J-B Weld (epoxy), Gorilla Glue (polyurethane), and Elmer’s Glue (polyvinyl acetate) were tested for the lap shear adhesion on Al, steel, and glass surface with (12 mm × 12 mm) 144 mm^2^ adhesive area, where two substrates were joined at room temperature (23°C) following the manufacturer’s recommended procedure. The adhesion results as shown in Table 1 and table S3 demonstrate that the SiNP S-Bpin composite-based adhesives exhibit significantly greater adhesion strength, compared to commercial adhesives and most of the reported adhesives with dynamic covalent bonds (Fig. 5H). Particularly, the adhesion property from the dry adhesive based on the 20 wt % SiNP S-Bpin composite widely exceeds these four commercial adhesives and also maintains very strong lap shear adhesion at 95°C on Al (8.66 MPa) and steel (11.4 MPa) surfaces (Fig. 5I and Table 1), which is about five times higher than that of the representative thermoset adhesive, J-B Weld of 1.73 MPa on Al and 2.04 MPa on steel surface. It should be noted that the other commercial adhesives including SEBS and Elmer’s glue could not be measured at 95°C because their lap shear adhesion strength at 95°C was too weak to be measurable. The high adhesion strength of SiNP S-Bpin composites at high temperature may open up many applications that require maintaining strong bonding at high temperature.

**Table 1.** Lap shear strength of commercial glues and 20 wt % SiNP S-Bpin composite.

**Samples**	**Temperature** **(°C)**	**Aluminum** **(MPa)**	**Steel** **(MPa)**	**Glass** **(MPa)**
20 wt % SiNPs (wet adhesive)	23	9.38 ± 1.39	9.83 ± 1.08	
20 wt % SiNPs (dry adhesive)	23	25.01 ± 0.92	28.54 ± 1.45	39.6 ± 3.2
Ethyl cyanoacrylate (Loctite Super Glue)	23	9.25 ± 0.96	17.6 ± 1.4	6.5 ± 1.2
Epoxy (J-B Weld)	23	7.55 ± 0.07	6.50 ± 0.50	9.40 ± 0.35
Gorilla Glue	23	10.1 ± 2.7	13.9 ± 0.2	6.60 ± 0.67
Elmer’s Glue All	23	2.73 ± 1.2	2.19 ± 0.5	Not tested
20 wt % SiNPs (wet)	95	4.13 ± 0.26	4.1 ± 0.28	Not tested
20 wt % SiNPs (dry)	95	8.66 ± 0.88	11.4 ± 1.01	Not tested
Epoxy (J-B Weld)	95	1.73 ± 0.05	2.04 ± 0.65	Not tested

The remarkably strong and tough adhesive with rebonding ability of multiphase composites indicates the great potential of the biomimetic strategy that is used for this study. In Fig. 5J, we proposed a plausible mechanism of adhesion for the SiNP S-Bpin composite adhesive, which has mussel-mimicking dynamic chemical (B-O bond) and physical bond (H-bond or van der Waals interaction) between the hydroxy-terminated substrates and adhesives. These dynamic physical and chemical bond exchanges facilitate the surface contact at macroscopic and microscopic scales, leading to the enhanced adhesive strength (*46*). Furthermore, the nacre-mimicking multiphase composite structure, including the presence of dynamic covalent interactions within the matrix and with the substrate surface, coupled with triblock architecture enabled this strong and tough adhesion. The high adhesion strength of S-Bpin composite relative to commercial adhesives also suggests the efficacy of biomimetic multiphase composites with dynamic reversible bonds as a new design of adhesive materials. While this study showed the method of both solution and dry melt adhesive application, melt adhesive usage with further tailoring the process temperature may lead to the rapid adoption of these reversible adhesives in the commercial sector.

### Adaptability with various fillers

This study has demonstrated that boronic ester on S-Bpin can be readily crosslinked with hydroxyls on the surface of various fillers; thus, the concept should be applicable beyond SiNPs. To further demonstrate the potentials of S-Bpin or boronic ester functionalized polymer in general, other fillers having hydroxyl groups on the surface were incorporated into S-Bpin. We synthesized four types of S-Bpin–based composites by dispersing 2.5 and 5 wt % of several micrometer-sized fillers, including 3M glass beads (diameter of 20 to 40 μm), glass fibers (diameter of 11 to 14 μm), cellulose microcrystals (20-μm size), and cellulose microfiber (medium size), into the THF solution (100 mg/ml) of S-Bpin. A limited amount of fillers was incorporated to fabricate these composites due to the inability to completely disperse fillers at higher loadings. Similar to the SiNPs, we expect that these fillers also form covalent crosslinking with S-Bpin via boron transesterification reaction, as they also have hydroxyl groups. The composites with these fillers also exhibited significantly higher tensile strength with slightly decreased elongation at break compared to that of S-Bpin (fig. S15, A and B). Following our optimized condition, the lap shear adhesion was measured on an Al substrate using both composite film and solution. The lap shear adhesion results (fig. S15, C and D) indicate that all the fillers exhibit strong adhesive property on Al surface, exhibiting 5 to 20 MPa, while the values were lower than those of SiNPs, probably due to the better dispersity and higher surface area of SiNP samples than the composites with micrometer-scale fillers. The high adhesion strength with different fillers proves wide adaptability of the concept, where the S-Bpin can form a dynamic covalent bond with various kinds of hydroxy-terminated fillers and substrates, leading to outstandingly strong and ductile reprocessable adhesive property.

## DISCUSSION

In this research, we demonstrated a biomimetic multiphase design strategy for preparing exceptionally tough adhesives with reprocessability. The incorporation of dynamic boronic ester–based functional groups into SEBS, a commodity thermoplastic elastomer, formed dynamic exchangeable covalent bonds with hydroxyls on the surface of diverse fillers and substrates, which were further confirmed by DFT calculations. The dynamic interaction between SiNPs and S-Bpin matrix reinforced the triblock copolymer network and significantly enhanced their tensile strength, toughness, and temperature service window while maintaining recyclability. Their mechanical robustness coupled with unveiled dynamic interaction with hydroxyl groups on various oxide surfaces led to enhanced adhesive strength, toughness, and debonding energy. Especially, 20 wt % SiNP S-Bpin composite provides the balance between cohesive and adhesive forces and exhibits remarkably strong adhesion and work of debonding with Al, steel, and glass surfaces. The unprecedented tough adhesion characteristics at both room and elevated temperature in this study surpass those of many existing commercial adhesives. The tailored design of the triblock copolymer coupled with dynamic boronic ester bonding to readily available hydroxyl fillers and surfaces reveals an attractive strategy for the future design of tougher materials and adhesives.

## MATERIALS AND METHODS

### Synthesis of S-Bpin

S-Bpin was synthesized by following the literature procedure (*47*). Briefly, SEBS (5.00 g, 14.24 mmol of styrene units), B_2_Pin_2_ (12.65 g, 49.8 mmol, 3.5 equiv), [IrCl(COD)]_2_ (0.502 g, 1.5 mol % based on the amount of B_2_Pin_2_), dtbpy (0.401 g, 3 mol % based on the amount of B_2_Pin_2_), anhydrous THF (50 ml), and a magnetic stirring bar were placed into a 100-ml flame-dried round-bottom flask and purged with argon for 30 min. The reaction flask was sealed under an argon atmosphere and placed in the preheated oil bath at 75°C. The reaction was stopped after 24 hours and cooled to room temperature. The solution was diluted with chloroform (25 ml) and precipitated into methanol, and the resulting white color polymer was collected to dry under vacuum at room temperature. The dissolution and precipitation methods were repeated two more times for the complete removal of catalysts and other unreacted small molecules. The degree of functionalization of styrene units was calculated from ^1^H NMR (fig. S1) based on the relative intensity of the methyl group in the 1,2-butylene unit of the polymer main chain (at 0.78 to 0.88 ppm) with respect to the increased integral ratio of the overlapping SEBS-methylene and boronated ester methyl resonance (at 0.9 to 1.5 ppm).

### Synthesis of SiNP S-Bpin composites and their crosslinked films

S-Bpin (1.0 g) was dissolved into anhydrous THF (12 ml) in an oven-dried vial equipped with a stir bar. The solution was filtered using a 0.45-μm pore size filter to remove undissolved artifacts. SiNPs in methyl isobutyl ketone (MIBK) solution were added into S-Bpin solution with continuous stirring. After 1-hour stirring at room temperature, solvents were dried under vacuum to give the crosslinked composite as a rigid solid. The composite product was further dried at 120°C under vacuum overnight to remove residual solvent. This partially cured composite was hot-pressed at 215°C for 3 hours with constant pressure to make the fully cured composite film after slow cooling.

### Synthesis of borylated polystyrene and SiNP-based composite

Borylated polystyrene was synthesized by following the same procedure of the synthesis of S-Bpin as shown in the reaction scheme (fig. S3A). ^1^H NMR (fig. S3B) demonstrated the borylated polystyrene product formation. The nanocomposite was also prepared from borylated polystyrene and SiNPs following the abovementioned procedure.

### Solvent resistance study

We performed solvent resistance analysis of the SiNP S-Bpin composites (fig. S8). Fully crosslinked composite films (15 mg) were subjected to different solvents (1 ml) such as THF, DCM, chloroform (CHCl_3_), DMF, and deionized water for 7 days, and their solubility was monitored at room temperature. After 7 days of immersion under solvent, the composite films underwent some degree of swelling in THF, DCM, and CHCl_3_ but did not dissolve completely (fig. S8A). These composite films are very stable underwater and DMF as they absorbed a negligible amount of water after 7 days. The water stability was further confirmed as they did not change their chemical compositions observed in TGA and FTIR (fig. S8, B and C).

### Lap shear adhesion

Lap shear adhesion measurements for Al and steel were conducted following a modified version of the ASTM D1002 method (ASTM D 1002-10) in an MTS Alliance RT/5 tensile frame equipped with a 5-kN load cell at a crosshead speed rate of 2 mm min^−1^. For solution adhesive, SiNP S-Bpin composite solution (200 μl) was spread onto the substrates using a syringe and dried for 1 min at room temperature. The adherents were overlapped (12 mm by 12 mm) in a single lap shear configuration (fig. S12A). The lap shear specimens were cured at 120°C overnight under a high vacuum and fully cured by hot pressing at 215°C for 2 hours under constant pressure (~0.096 MPa). The samples were cooled down to room temperature before performing the test. For dry adhesive (melt adhesive), fully cured SiNP S-Bpin composite film was cut into small pieces with an area of (3 mm × 3 mm) 9 mm^2^ or (6 mm × 6 mm) 36 mm^2^ and placed between two overlapping substrates. These overlapping substrates were placed on hot press at 215°C and pressed for 2 hours under constant pressure. After cooling at room temperature, the lap shear strength was measured and the average results of at least three specimens were reported with error bars of SD. The commercial adhesive samples were prepared following the manufacturer’s instructions. The adhesive performance at elevated temperature was conducted on Al and steel at 95°C. The lap shear specimen was heated under a high temperature–controlled system, and the lap shear test was performed when the temperature was stabilized at 95°C. For glass substrates, the lap shear adhesion measurements were performed in an MTS Alliance RT/5 tensile frame equipped with a 2-kN load cell at a crosshead speed rate of 2 mm min^−1^. Following a similar procedure, the lap shear adhesion test was performed for the glass substrates.

Lap shear adhesion is defined as the maximum force (in newtons) of the adhesive joint obtained from the lap shear test divided by the overlap area (in square millimeters) of adhesives.

$$\text{Lap shear strength}=\frac{\text{Force}\;(N)}{\text{Adhesive area}\;(mm^{2})}$$

Work of debonding is defined as the integrated area under the force-versus-extension curve. The integration was achieved using the Integrate feature OriginPro software, version 2020.

### DFT calculations

DFT calculations were used to explore boron ester binding on silica, alumina, and iron oxide hydroxylated surfaces, as these are appropriate models for understanding the chemical interactions with Si-O-H, Al-O-H, and Fe-O-H groups when chemical bonding is the primary energy of contribution. A detailed explanation of DFT calculations (*31*, *57*, *59*–*65*) is given in the Supplementary Materials.
