# Supplementary material for: Design of tough adhesive from commodity thermoplastics through dynamic crosslinking
Source: Sci Adv. 2021 Oct 15;7(42):eabk2451. doi: 10.1126/sciadv.abk2451 (PMC8519568; doi:10.1126/sciadv.abk2451)
Supplement: Supplementary file 1 — Supplementary Text Figs. S1 to S18 Tables S1 to S6 [file sciadv.abk2451_sm.pdf]

Science Advances
AAAS

Supplementary Materials for

Design of tough adhesive from commodity thermoplastics through dynamic crosslinking

Md Anisur Rahman, Christopher Bowland, Sirui Ge, Shree Ram Acharya, Sungjin Kim, Valentino R. Cooper, X. Chelsea Chen, Stephan Irle, Alexei P. Sokolov, Aditya Savara, Tomonori Saito*

*Corresponding author. Email: saitot@ornl.gov

Published 15 October 2021, *Sci. Adv.* **7**, eabk2451 (2021)
DOI: 10.1126/sciadv.abk2451

This PDF file includes:

Supplementary Text
Figs. S1 to S18
Tables S1 to S6

Supplementary Materials

Supplementary Text

Materials. Polystyrene-*b*-poly(ethylene-*co*-butylene)-*b*-polystyrene (SEBS) (M_n = 118 kg/mol with Đ = 1.08; 18 mol % polystyrene repeating unit; 30 wt% polystyrene), 4,4′-di-tert-butyl bipyridine (dtbpy) and chloro(1,5-cyclooctadiene)iridium(I) dimer ($[IrCl(COD)]_2$) were purchased from Sigma-Aldrich. Bis(pinacolato)diboron (B_2Pin_2) was purchased from oakwood chemicals. Colloidal silica nanoparticles (MIBK-ST, 10-14 nm) mono-dispersed in methyl isobutyl ketone (MIBK) was donated by Nissan chemicals. Cellulose microcrystalline (20 µm), and cellulose microfiber (medium size) were purchased from Sigma-Aldrich. 3M™ Glass Bubbles K46 were obtained from 3M. Glass fibers (11-14 µm) were obtained from Owens Corning Composite Materials. Anhydrous tetrahydrofuran (THF) was purchased from Acros Organics and used as received. Methanol, dichloromethane (DCM), chloroform ($CHCl_3$), and dimethylformamide (DMF) were reagent grade and used without further purification.

Characterization Methods.^{1}H NMR spectra were recorded with a Bruker instrument (400 MHz) and signals were measured relative to the residual chloroform signal from $CDCl_3$ for ^{1}H at 7.26 ppm. Infrared (IR) spectra were recorded on a Cary 600 Series FT-IR Spectrometer (Agilent Technologies). Thermogravimetric analysis (TGA) of the composite samples and model compound was performed on TGA Q50, TA Instrument. Approximately 10 mg of each completely dried sample was weighed out to test TGA. The samples were heated up to 800 °C from room temperature at a heating rate of 10 °C min^{-1} under a dry nitrogen atmosphere (flow rate: 40 mL min^{-1}).

Tensile Analysis. Tensile stress and strain of polymer composite samples were measured using an Instron 3343 universal testing system equipped with 1 KN sensor following the ASTM D1708

standard. A punch was used to cut the dog-bone shape films with a width of 5 mm, a length of 22 mm, and an average thickness of 0.5 mm, which were tested at room temperature. Samples were elongated at the rate of 1 mm s^{-1} till break. Toughness was calculated from the area under the stress−strain curve. Youngs modulus was measured at 1 % strain of stress-strain curve. The mechanical properties were reported from the average of at least three specimens for each sample.

Dynamic Mechanical Analysis (DMA). Dynamic mechanical properties were carried out by TA Instruments RSA-G2 Solids Analyzer using a tension clamp. Samples were prepared by hot pressing at 215 °C, and films were cut into a rectangular shape. Samples were tested in a temperature range from -120 °C to 300 °C at a rate of 3 °C min^{-1} with a frequency of 1 Hz and 15 µm amplitude.

Rheology Measurements. Rheological measurement was carried out by an AR2000ex rheometer (TA Instruments) using 8 mm plates with a parallel plate geometry. For stress relaxation experiments, the samples were tested in a temperature range from 230 °C to 270 °C by applying a constant strain at 2 % and allowed to relax for 20 min. Before each measurement, the sample underwent thermal stabilization for 5 min to make sure that thermal equilibrium was reached. The relaxation modulus was monitored over time. The relaxation times were determined from a plot of the normalized relaxation modulus vs time when the normalized relaxation modulus reaches 1/e (37%). The apparent activation energy (E_a) was measured from the fitted curves of ln (τ) vs 1000/T according to the Arrhenius equation.

Transmission Electron Microscopy (TEM). To prepare TEM samples, thin sections with a thickness of about 100 nm were obtained by cryomicrotoming a piece of bulk film at −80 °C using a Leica EM FC7 cryomicrotome. The thin sections were placed onto a lacey carbon-supported copper grid (Electron Microscopy Sciences). The samples were not stained. TEM experiments

were performed on an aberration-corrected FEI Titan S 80-300 TEM/STEM microscope, using 300 keV acceleration voltage.

Small Angle X-ray Scattering (SAXS). The SAXS measurement was conducted on XEUSS 3.0 (Xenocs, France) equipped with a Cu Kα microfocus source and a Pilatus 300 k detector (Dectris, Switzerland). The scattering vector (q) was calibrated by a silver behenate standard material. The distance between sample and detector was 0.9 m. The sample holder was made of stainless steel, on which there are holes to place the samples. The sample was made into a thin film around ~500 μm and fixed on the sample holder, covering the whole.

Preparation of tough adhesive. SiNP S-Bpin composite can be applied in two different states, solution adhesive and dry adhesive (melt adhesive).

Solution adhesive. The adhesive solution was prepared by dissolving S-Bpin (0.5 g) into anhydrous THF (5 mL) in a dry vial equipped with a stir bar and the solution was filtered with a 0.45 μm filter. A respective amount of SiNPs in MIBK solution was added into S-Bpin solution with continuous stirring for 1 h at room temperature and directly applied to measure the adhesive property.

Dry adhesive (melt adhesive). Fully cured SiNP S-Bpin composite film was directly applied as dry adhesive. The composite film was cut into a small area, then put between the two overlapping substrates and hot-pressed at 215 °C for 2 h.

Preparation of substrates for lap shear testing. Aluminum (Al) and steel substrates were prepared for lap shear testing by cutting each material into rectangular pieces with a dimension of 8.89 cm long × 1.25 cm wide × 0.16 cm thickness. Al (6061 T6) and steel (A36) sheets were purchased from McMaster-carr and cut into rectangular pieces from the ORNL machine shop. Each substrate was drilled to make a centered hole from 1 cm of one end with a 0.64 cm diameter.

Substrates were sanded with 50 grit sandpaper and washed with dilute acid and base solutions, deionized water, acetone, methanol, and THF before an adhesion test. Glasses with a custom dimension of 8.89 cm long × 1.5 cm wide × 0.31 cm thickness were purchased from Cincinnati Gasket & Industrial Glass. Glass was cleaned and washed with dilute acid and base solutions, deionized water, acetone, methanol, and THF. To further clean the glass surface, Corona treatment was applied to remove all the artifacts.

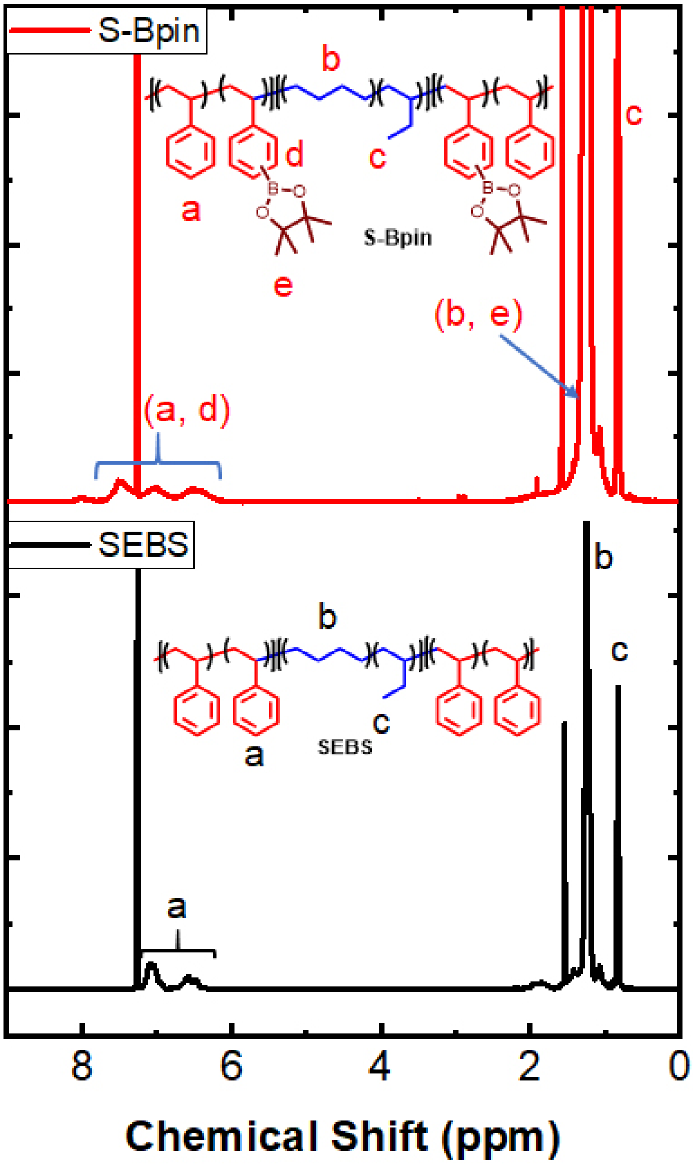

Fig. S1. **^{1}H NMR (400 MHz, CDCl3) of SEBS (black) and S-Bpin (red) triblock copolymer**.

The degree of functionalization was calculated following a literature procedure (1) based on the relative intensity of the methyl group in 1,2-butylene unit of the polymer chain (peak c) with respect to the increased integral ratio of the overlapping SEBS-methylene and Bpin methyl resonance (peaks b and e at 0.9−1.5 ppm). It was observed that 95% of original styrene (30 mol %) was borylated. The concentration for both SEBS and S-Bpin (20 mg/0.6 mL) in $CDCl_3$ was kept constant during the measurement.

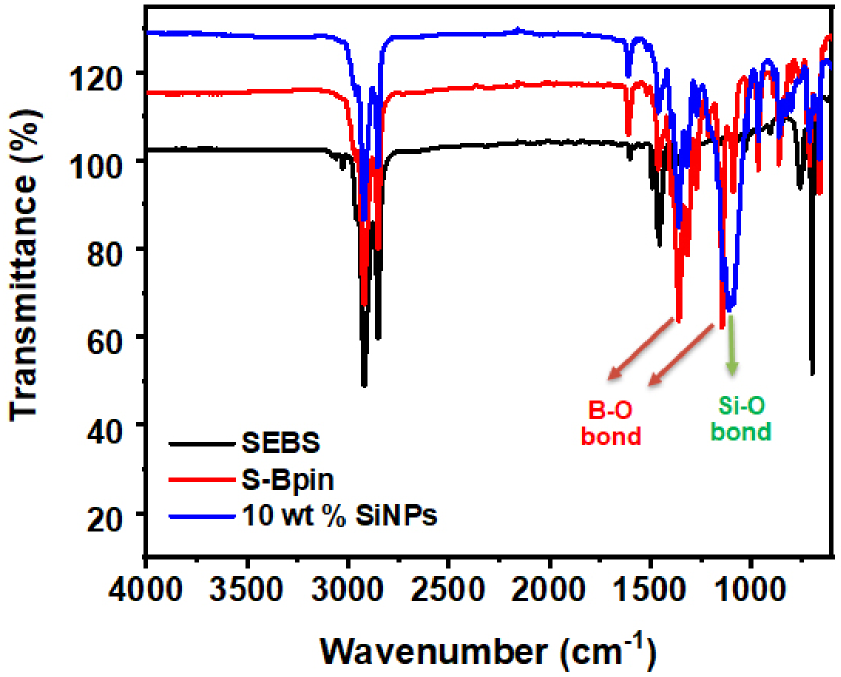

Fig. S2. **FTIR spectra of SEBS, S-Bpin, and 10 wt% SiNP S-Bpin composite**. The FTIR signals at 1350 cm^{-1} and 1123 cm^{-1} indicate asymmetric and symmetric stretching of the B-O bond. The peak at 1100 cm^{-1} is the characteristic absorption peak for Si-O bond in 10 wt% SiNP S-Bpin composite.

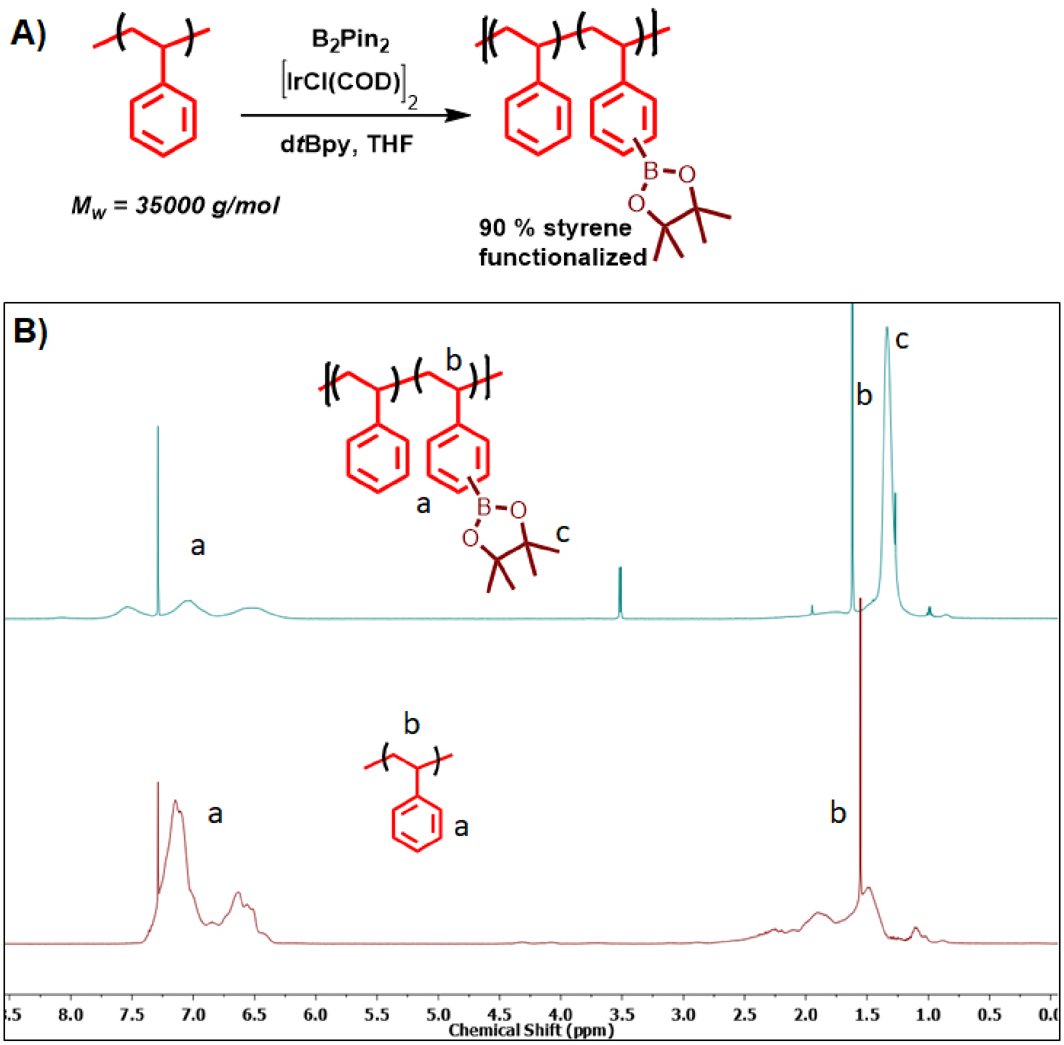

Fig. S3. **Synthesis and characterization borylated polystyrene homopolymers using** ^{1}**H NMR**. (**A**) Synthesis of borylated polystyrene homopolymers, (**B**) ^{1}H NMR (400 MHz, $CDCl_3$) of polystyrene (bottom) and borylated polystyrene (top) homopolymer.

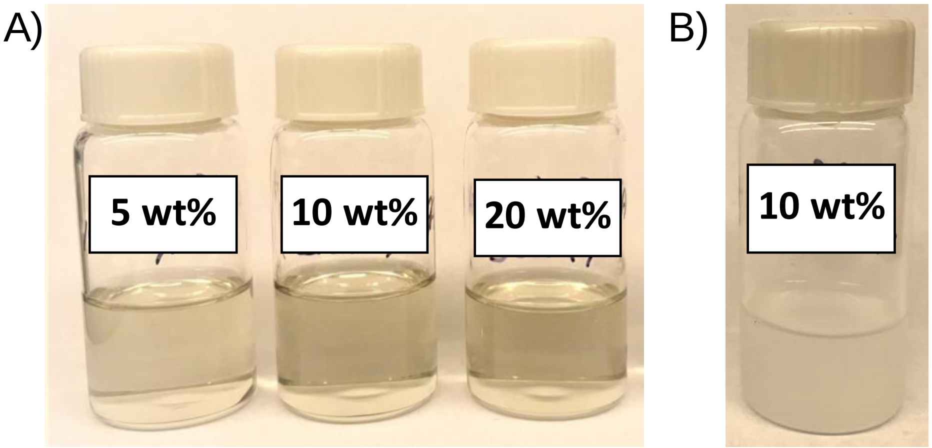

Fig. S4. **Dispersion of SiNPs analysis**. (**A**) 5-20 wt% SiNP S-Bpin composites dispersed in THF to form a clear solution (concentration 100 mg/mL), (**B**) SEBS and 10 wt % SiNPs form a cloudy solution in THF. Photo Credit: Md Anisur Rahman, ORNL.

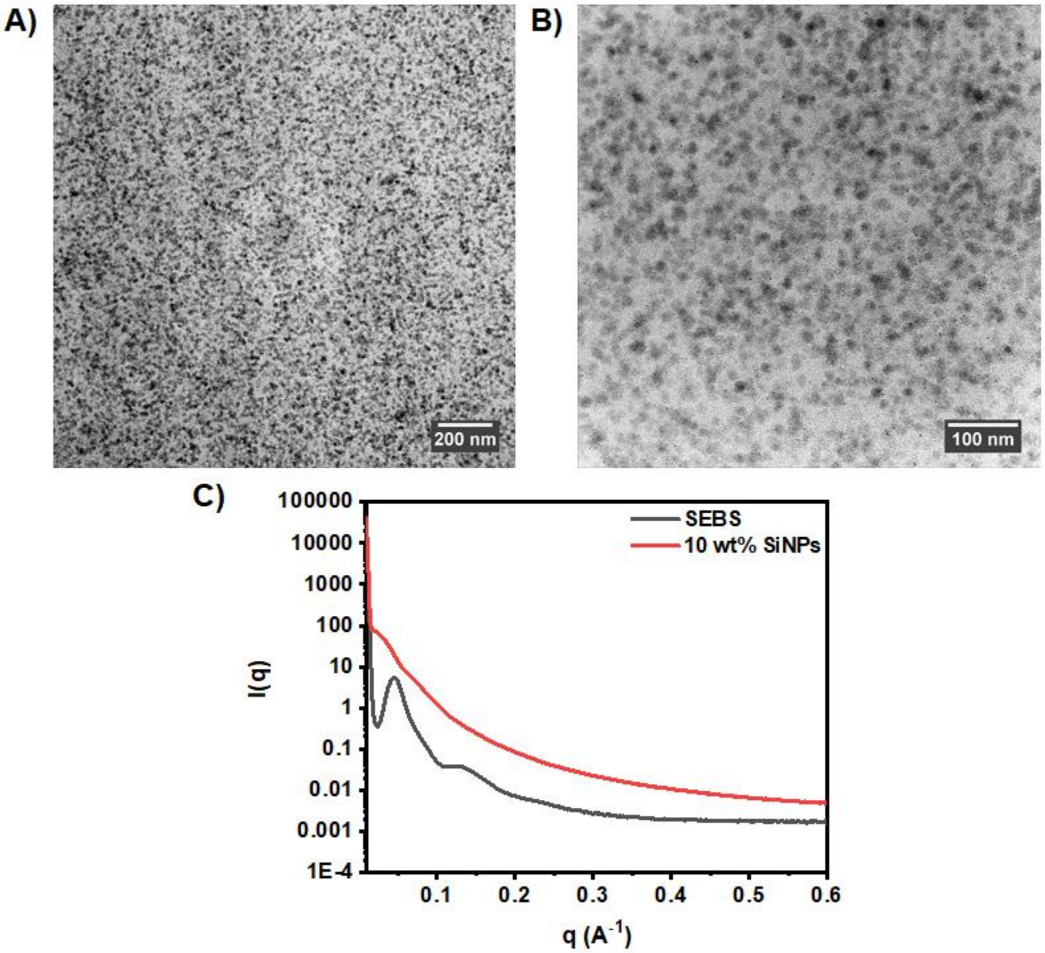

Fig. S5. **TEM images of 10 wt% silica loaded SiNP S-Bpin composite**. (**A**) TEM images with lower magnification, (**B**) TEM images with higher magnification, (**C**) SAXS profile of SEBS and 10 wt% SiNP S-Bpin composite at room temperature. Both SEBS and 10 wt% SiNP S-Bpin composite showed strong primary scattering peak, which confirms their microphase separation. The domain spacing of 10 wt% SiNP S-Bpin composite (d-spacing ~ 30 nm) is almost doubled compared to that of SEBS (d-spacing ~ 14 nm).

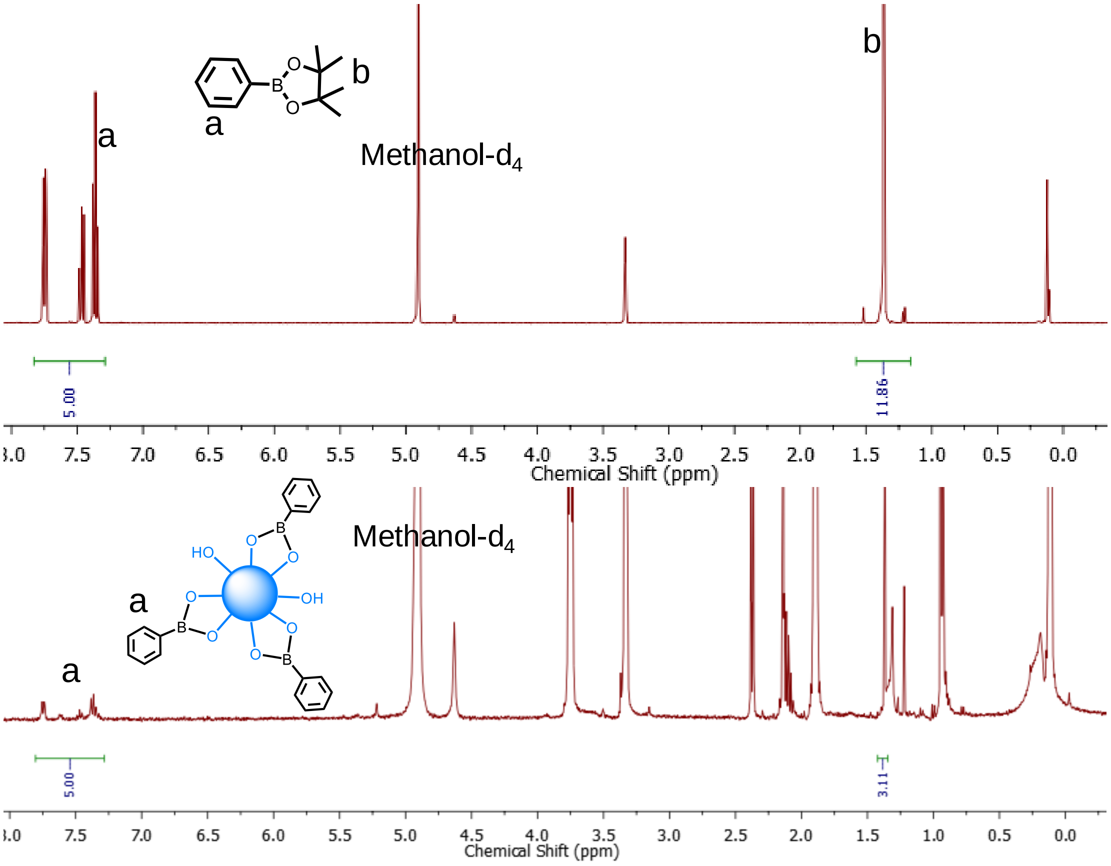

Fig. S6. **^{1}H NMR analysis of the model compound**. ^{1}H NMR (400 MHz, Methanol-d_4) of phenyl penacholester and model compound.

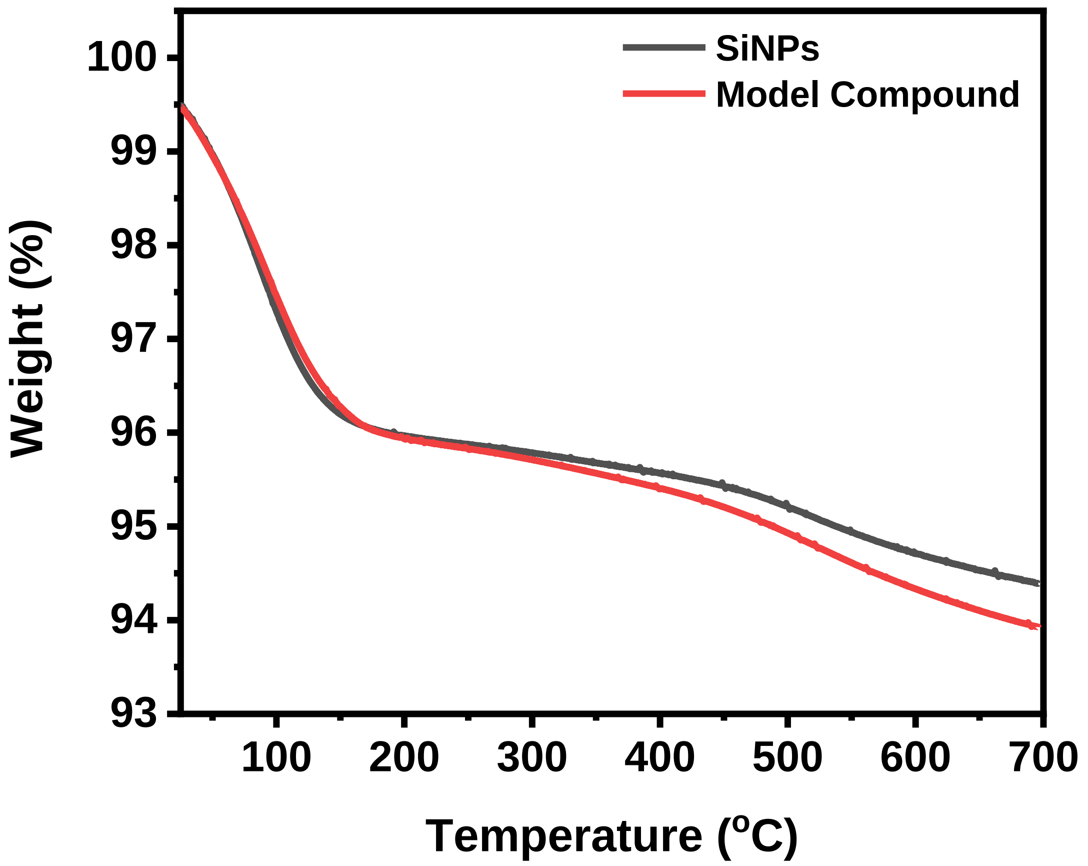

Fig. S7. **TGA curves of the model compound**. TGA curves of the model compound and SiNPs represent the successful modification of SiNPs surface.

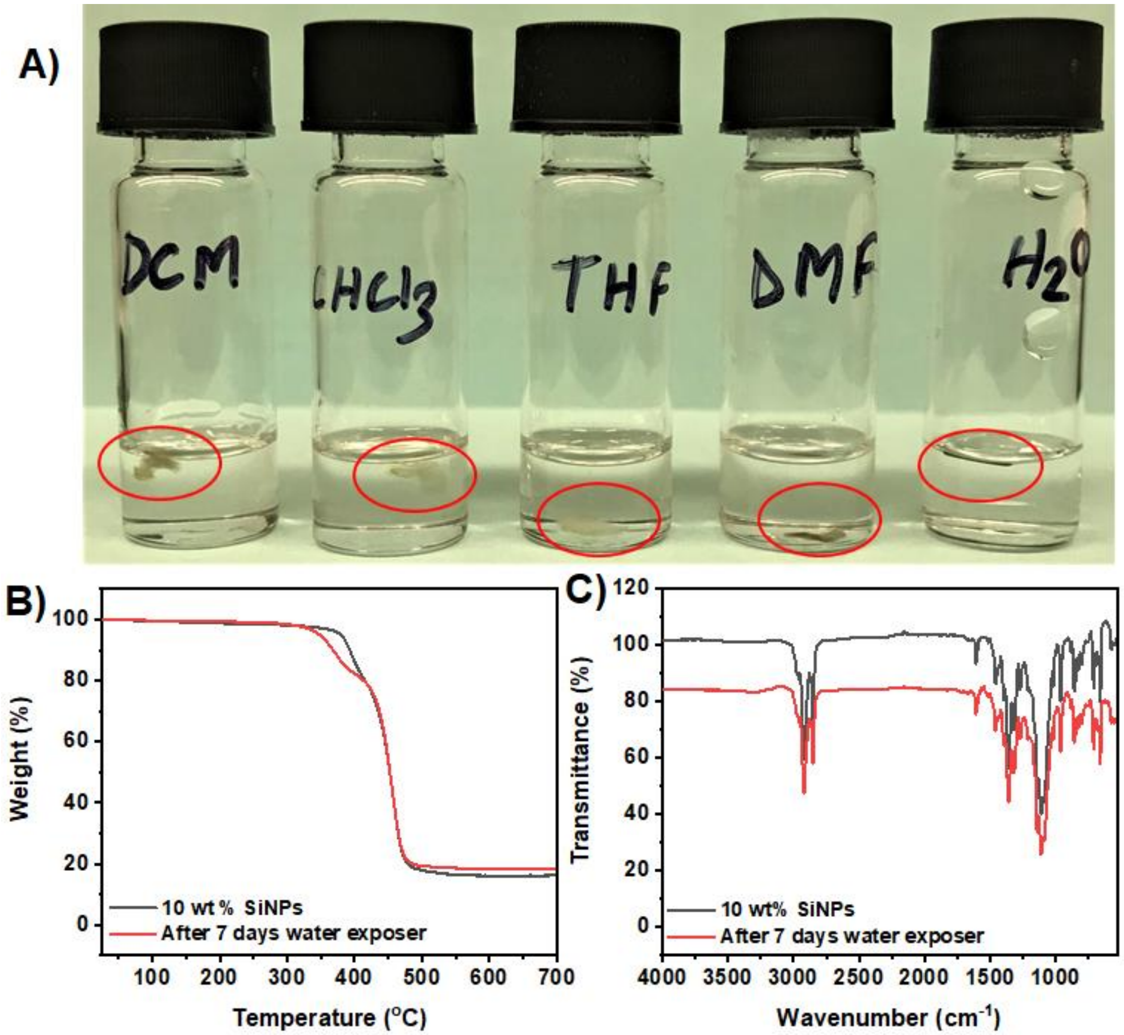

Fig. S8. Investigation of solvent resistance of SiNP S-Bpin composites. (**A**) Samples were immersed under different solvents for 7 days at room temperature and monitored for swelling, (**B**) TGA curve to check the water stability of this composite; TGA was measured before and after the sample was immersed under DI water for 7 days and compared, (**C**) FTIR spectra to investigate the chemical stability of composite sample before and after 7 days water exposure. Photo Credit: Md Anisur Rahman, ORNL.

Table S1. Thermal and mechanical properties of SEBS, S-Bpin, and different silica loaded composites.

Crosslinked polymer	T_g of Styrene block (ºC)	Ultimate tensile stress (MPa)	Strain at break (%)	Young's Modulus (MPa)	Toughness (MJm^{-3})
**SEBS**	90	22.37 ± 2.86	831 ± 98.99	14.5 ± 0.7	56.5 ± 0.90
**S-Bpin**	197	22.28 ± 2.42	576.5 ± 64.35	151.5 ± 4.95	56.3 ± 1.65
**5 wt% SiNPs**	204	33.0 ± 1.4	536.5 ± 51.62	249.5 ± 13.44	80.4 ± 2.85
**10 wt% SiNPs**	207	39.5 ± 3.53	494 ± 14.14	288 ± 9.9	91.5 ± 4.23
**20 wt% SiNPs**	211	29.0 ± 2.83	455 ± 11.31	368 ± 42.43	62.6 ± 3.58
**30 wt% SiNPs**	212	26.5 ± 0.71	267.5 ± 41.72	464 ± 35.36	40.0 ± 1.80

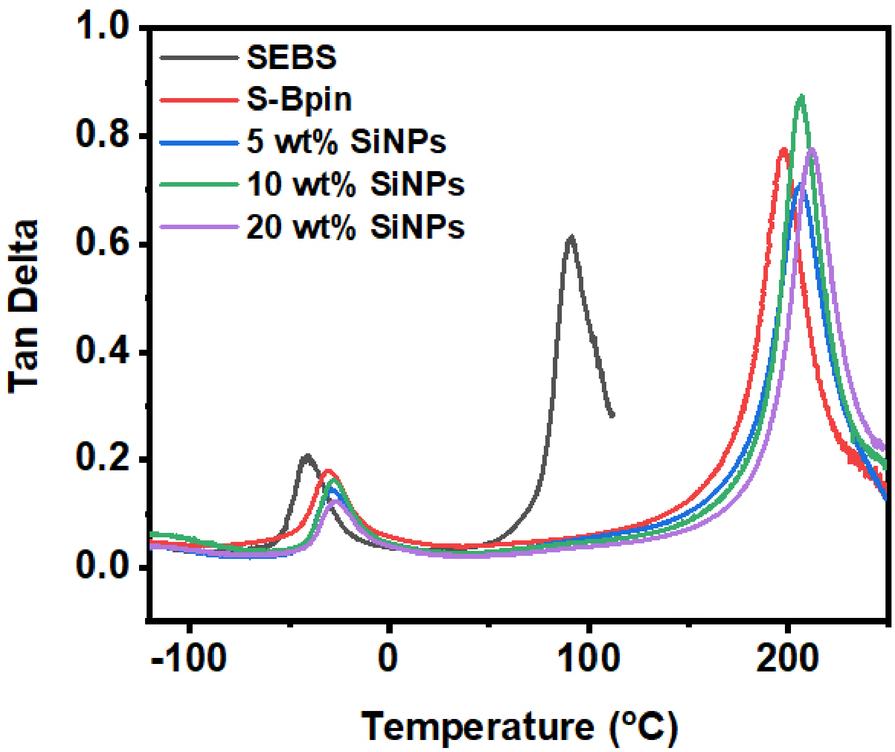

Fig. S9. **Tan delta plot obtained from DMA to determine glass transition temperature and thermomechanical properties.** This plot shows SEBS, S-Bpin, and SiNP S-Bpin composites have two glass transition temperatures.

Table S2. **The stress relaxation times of 10 wt% and 20 wt% SiNP S-Bpin samples at different temperatures**.

Samples	Relaxation time, τ (s)				
	230 °C	240 °C	250 °C	260 °C	270 °C
**10 wt% SiNPs**	127	74	35	12	
**20 wt% SiNPs**		415	300	142	60

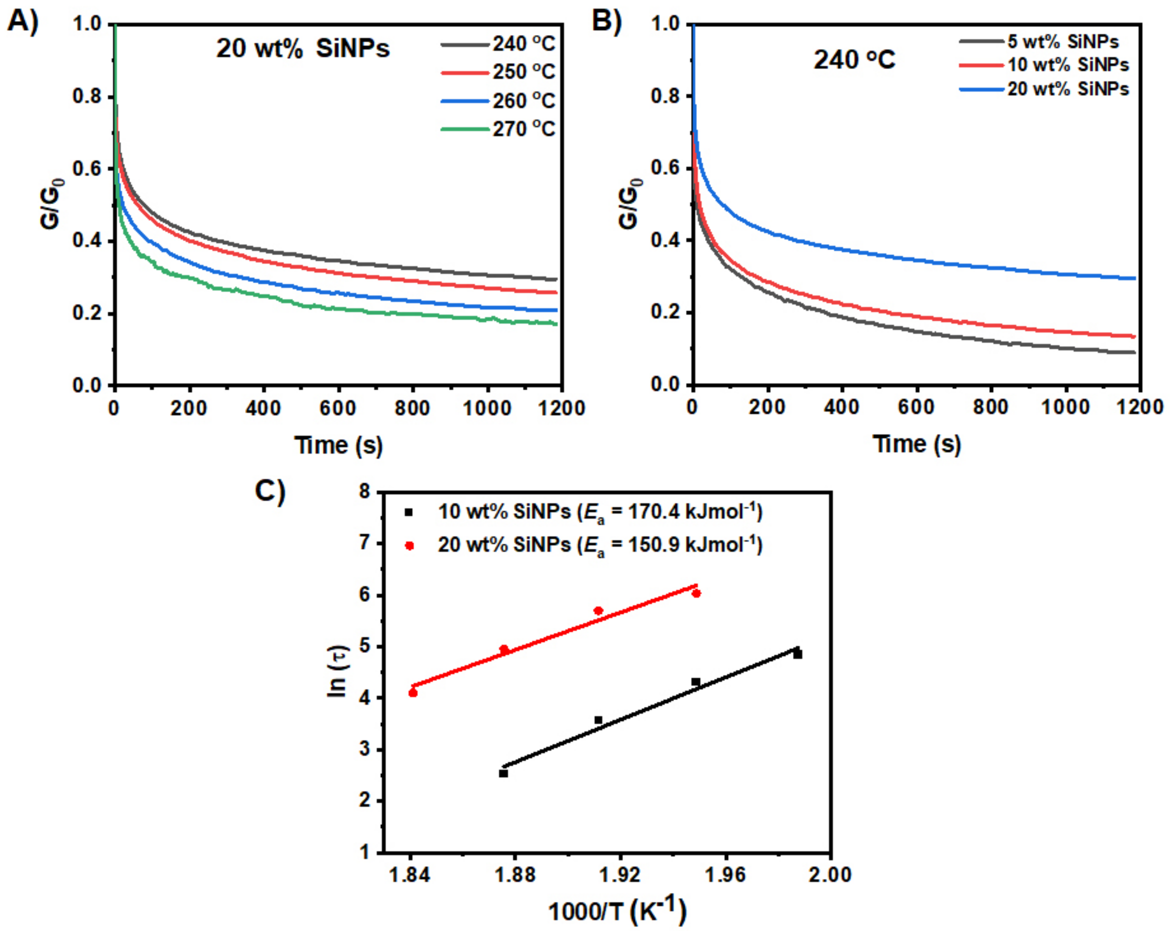

Fig. S10. Stress-relaxation of SiNP S-Bpin composites using 2 % strain. (**A**) Normalized stress relaxation of 20 wt% SiNP loaded composites at temperature range from 240 to 270 °C, (**B**) Stress relaxation behavior of 5 wt%, 10 wt%, and 20 wt% SiNP S-Bpin composites at 240 °C, (**C**) Plots for ln (τ) versus 1000/T of 10 wt% and 20 wt% SiNP S-Bpin composites. The characteristic relaxation time is obtained from the normalized stress relaxation curves and it is denoted as τ, the time needed to reach the normalized modulus to 1/e (37%) from the initial value.

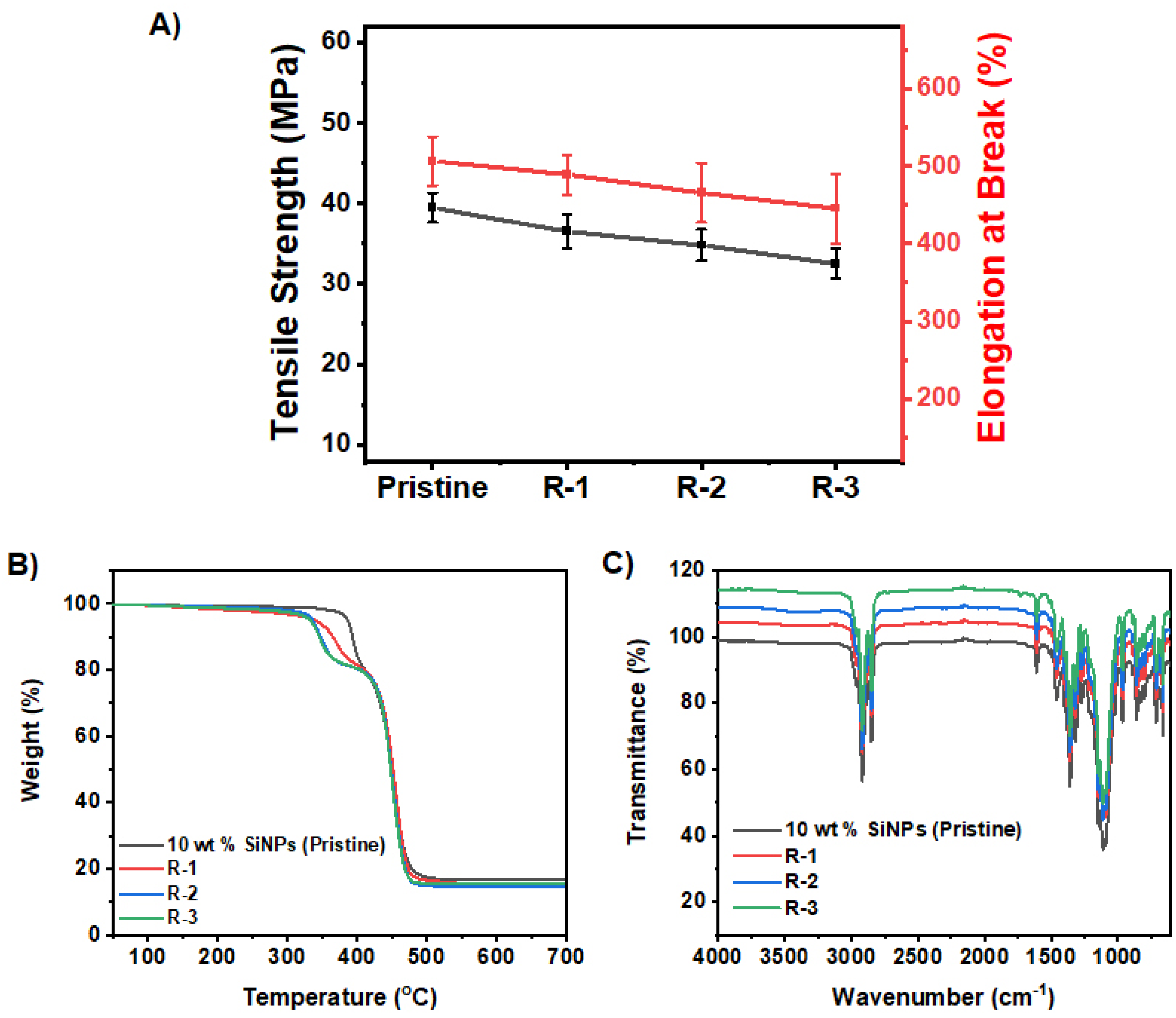

Fig. S11. Reprocessability of SiNP S-Bpin composites. (**A**) Average tensile stress and strain properties of reprocessed 10 wt% SiNP loaded samples, (**B**) TGA curves for the reprocessed 10 wt% SiNP loaded samples, (**C**) FTIR spectra of reprocessed 10 wt% SiNP loaded samples.

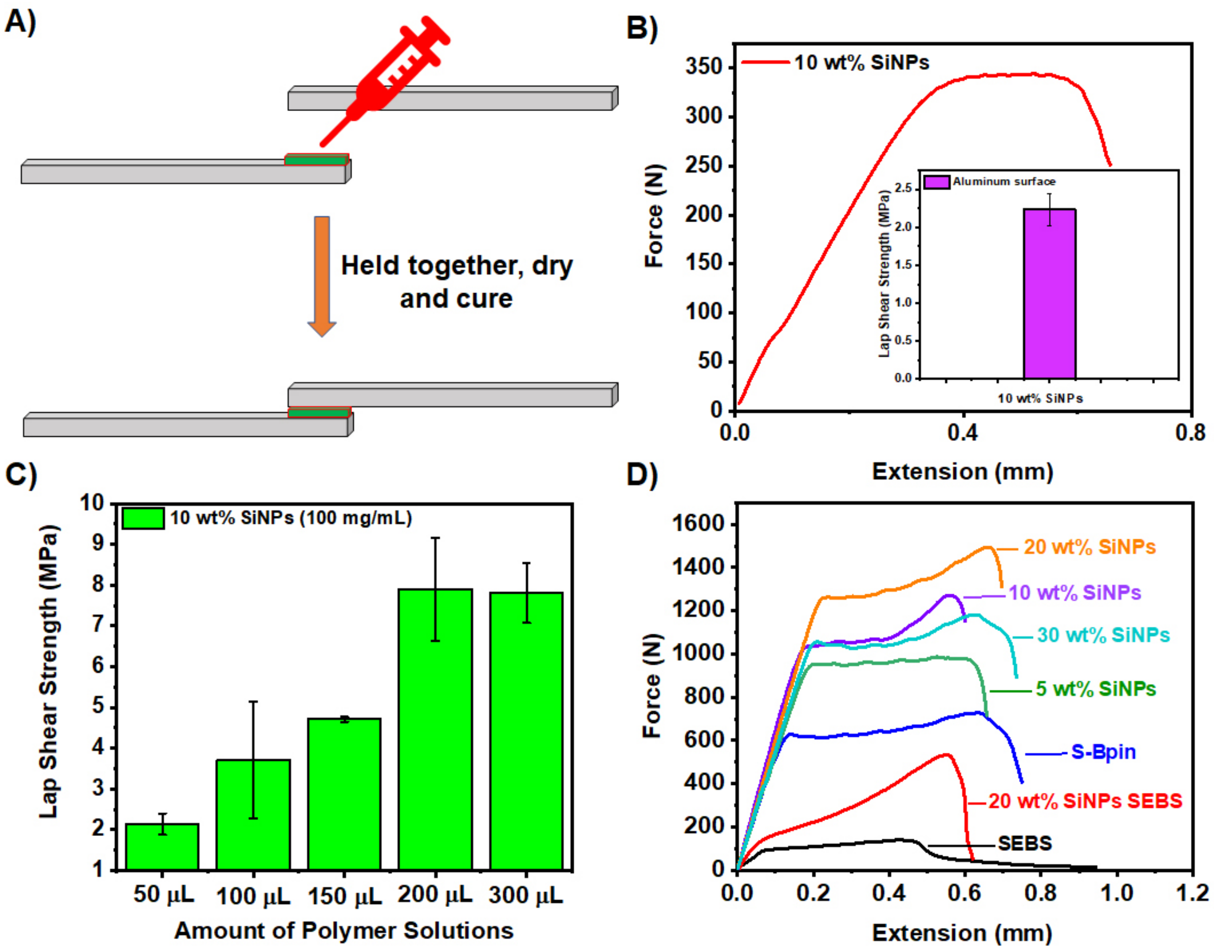

Fig. S12. Lap shear adhesion test using Al substrate. Lap shear adhesion was measured using (12 mm × 12 mm) 144 mm^2 adhesive surface area. (**A**) Schematic representation of lap shear adhesion testing setup, (**B**) Force-versus-extension curve of partially cured 10 wt% SiNP composites, (**C**) The investigation of the amount of polymer solution on lap shear adhesion using 10 wt % SiNP composite solution, (**D**) Force-versus-extension curves for different SiNP loaded samples on Al substrates.

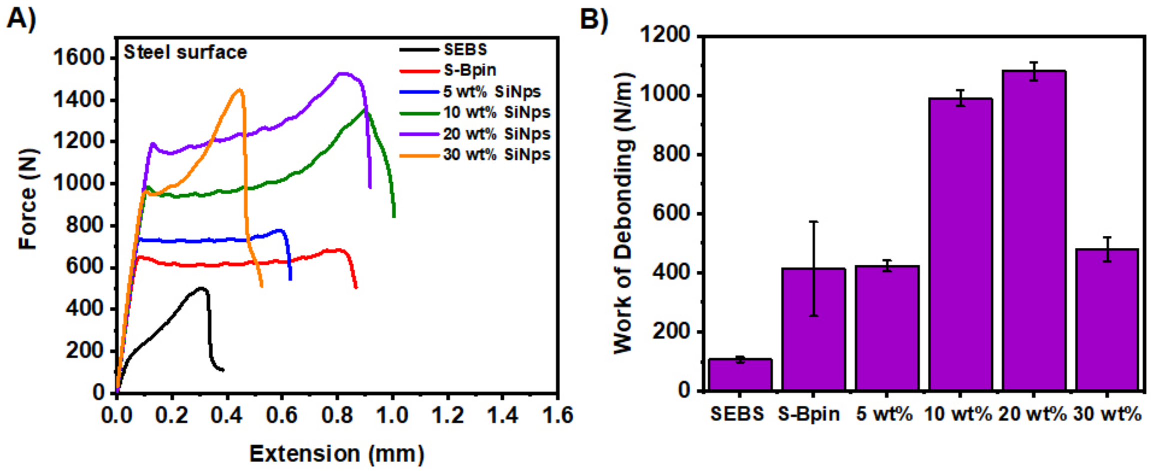

Fig. S13. Lap shear adhesion test using steel substrate. Lap shear adhesion was measured using (12 mm × 12 mm) 144 mm^2 adhesive surface area. **(A)** Force-versus-extension curves for different SiNP loaded samples on steel substrates, (**B**) Work of debonding for different amount of SiNP loaded composites on steel substrates.

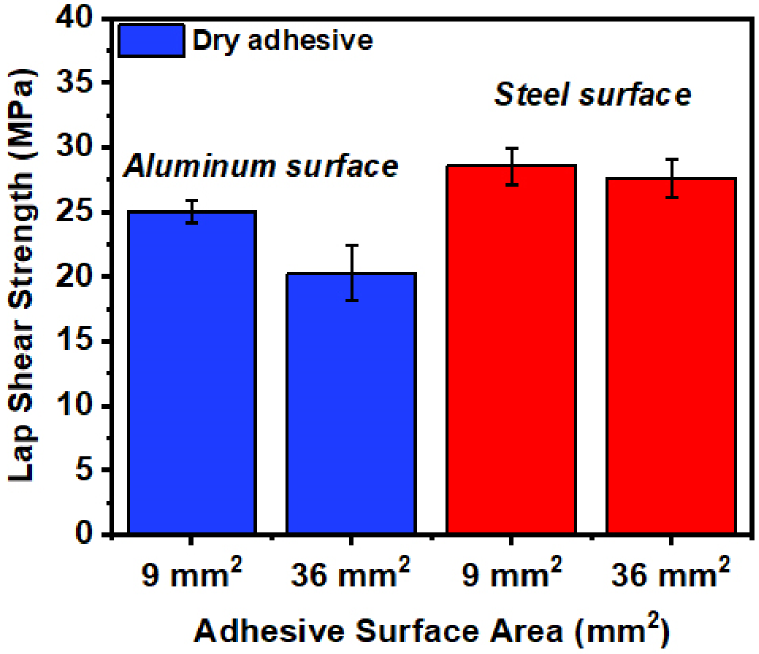

Fig. S14. Lap shear adhesion of composite films with different adhesive areas. Lap shear adhesion was measured using (3 mm × 3 mm) 9 mm^2 and (6 mm × 6 mm) 36 mm^2 on Al and steel substrates.

To understand the impact on adhesive behavior of SiNP S-Bpin composite film with a smaller area, a 9 mm^2, and 36 mm^2 composite adhesive film with 20 wt% SiNP S-Bpin was applied to Al and steel substrates and the lap shear strength was measured by the identical procedure (**Fig. S14)**. The measured adhesion is almost similar for both surface areas which is consistent with others. The adhesion for dry adhesive is higher than the adhesion measured using a composite solution, which is consistent with the literature (*37*) and may be due to the uniform surface coverage by the adhesive composite film, compared to solution casting, where the drying process might create unavoidable voids.

Table S3. **Lap shear adhesion of 20 wt% SiNP S-Bpin and relevant literature reported adhesives**. References (*2-14*) for the comparison of 20 wt% SiNP S-Bpin (dry adhesive) and relevant adhesive properties on different substrates.

Reference	Aluminum Surface (MPa)	Steel Surface (MPa)	Glass Surface (MPa)
20 wt% SiNPs (Dry Adhesive) (This work)	25.01 ± 0.2	28.54 ± 1.45	39.6 ± 3.2
Ref. 5	2.6 ± 0.3		
Ref. 7	11.0 ± 0.5	10 ± 1	
Ref. 23		23 ± 1	51 ± 3
Ref. 37	8.5		
Ref. 38	0.52		
Ref. 39	1.5 ± 0.03		
Ref. 40		6	5.3
Ref. 41		1.0	2.6
Ref. 42	7.5		
Ref. 43	23.7		
Ref. 44	6.5		
Ref. 45	3.42		

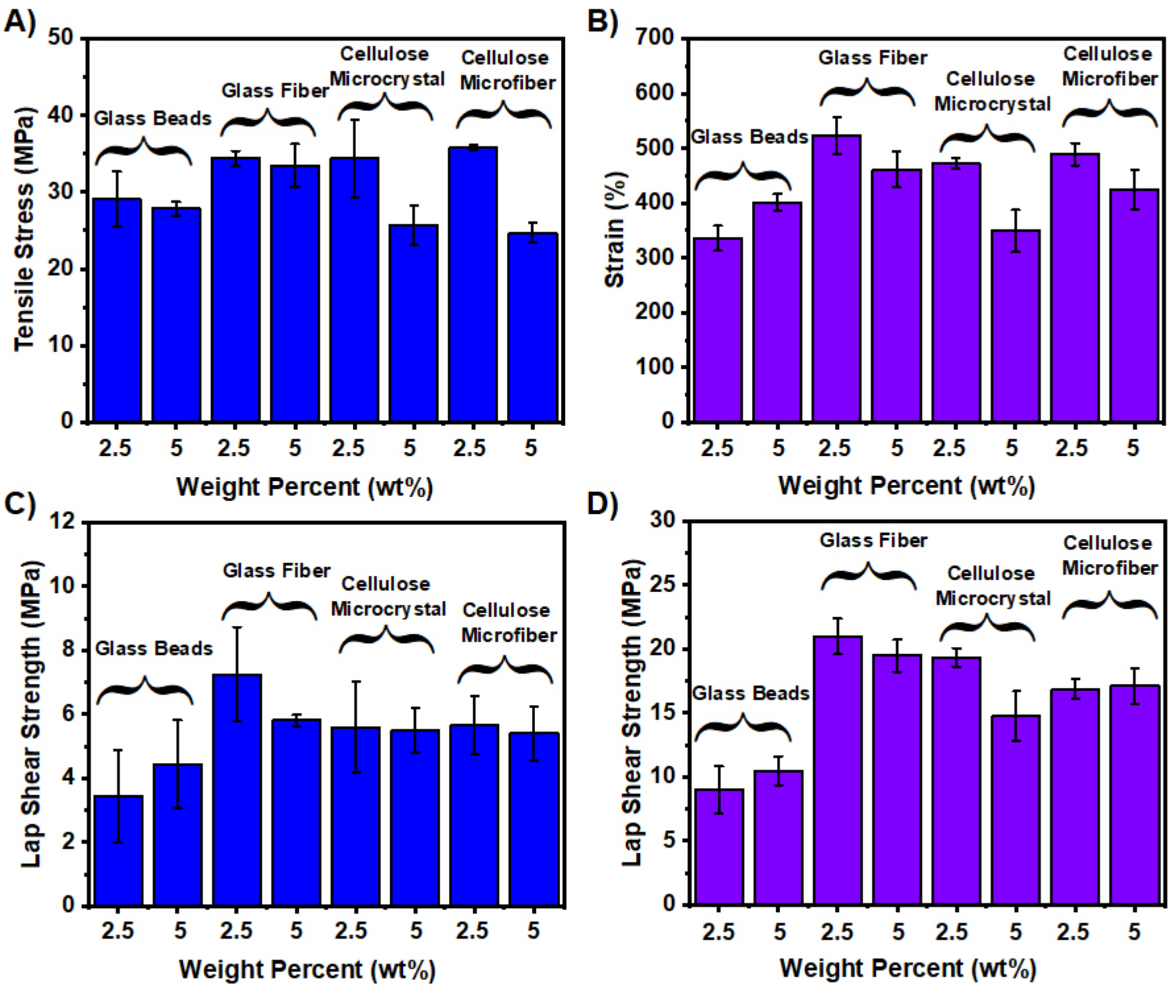

Fig. S15. Adaptability of different filler with S-Bpin. (**A**) Tensile stress of 2.5 and 5 wt % of different filler containing S-Bpin composites, (**B**) Strain of 2.5 and 5 wt % of different filler containing S-Bpin composites, (**C**) Lap shear adhesion of S-Bpin with different filler on Al using solution adhesive with an adhesive area of (12 mm × 12 mm) 144 mm^2, (**D**) Lap shear adhesion of S-Bpin with different filler on Al using dry/film adhesive with an adhesive area of (6 mm × 6 mm) 36 mm^2.

Density Functional Theory (DFT) Calculations

DFT calculations were utilized to explore boron ester binding on a silica, alumina, and iron oxide hydroxylated surfaces as these are appropriate models for understanding the chemical interactions with Si-O-H, Al-O-H, and Fe-O-H groups when chemical bonding is the primary energy of contribution. During this binding, the metal oxide termination begins with M-O-H terminations (where M is a lattice cation) and a boron ester reacts such that one or more M-O-H moiety is converted to Si-O-B moieties. Such possibilities are shown in **Fig. S16 and fig. S17**. As seen, we considered the case of an aryl-diboroxy/pinacoxy ester (B-Aryl-Pinacol), labeled as State 1 in **Fig. S16**, as well as the corresponding arylboronic acid itself (B-Aryl-OH), labeled as State 5 in **Fig. S16**. The latter structure has two OH groups in place of where the pinacol-type moiety was originally located when the polymer was synthesized. DFT calculations were conducted to assess the binding strengths of the boron ester moieties to a metal oxide surface: both for a mono-dentate product and for a bi-dentate product, where the molecule bonds to the surface via either one oxygen atom or via two oxygen atoms. The mono-dentate binding (State 2) was found to be more energetically favored than the bi-dentate binding (State 3). States 3 through 5 in **Fig. S16** were included to explore the possibility of water or hydroxyl groups affecting the binding. It was found that reaction with water was unfavored, and even if such reaction occurred, the most stable result would remain a mono-dentate bound polymer, as explained further below. Surface hydroxyls and surface protons could facilitate such reactions with the same stoichiometry as water, without any explicit water molecules being present or created.

In all structures case, we included an iso-propyl group in para-position of the benzene ring to represent the scenario where the boron ester is part of a polymer chain (initially with a pinacol-type moiety). The binding energies were computed using density-functional theory (*59, 60*) as

implemented in the Vienna Ab Initio Simulation Package (VASP) code (*61*). The generalized gradient approximation (GGA) parametrized by Perdew-Burke-Ernzerhof (PBE) (*62*) is used for the exchange-correlation (XC) functional in the DFT approach. The projected augmented wave (PAW) (*63, 64*) method and corresponding pseudopotentials are used to treat the electron-core interactions, and electronic wavefunctions are expanded using a plane-wave basis with a kinetic energy cutoff of 650 eV. The surface model consists of the six double layers of SiO_2 (101) with 4×6 Si atoms on each layer of which the bottom two double layers are fixed and the top and bottom layers are saturated with OH-groups. The Γ-point sampling of Monkhorst-Pack set of k-points (*65*) is used for the Brillouin zone integration due to the large size of the supercell. The optimization process allows the nuclear positions as well as the size/shape of the simulation box to change with force and energy convergence criteria of 0.05 eV/Å and 10^{-5} eV, respectively. The aluminum surface is modeled as the hydroxylated γ-Al_2O_3 (001) surface as described in (*31*) which is repeated 4×4 on lateral dimension and kept vacuum space of 20 Å to avoid interaction between periodic images of the adsorbed molecules. The model has OH adsorb on the surface Al, H on the surface O which are bonded only with surface Al and no adsorbate on the surface O which are bonded with Al on the layer below. Our model structure contains one less adsorbed H with respect to the reported model structure for stochiometric reasons: optimization of our model structure converged to the desired accuracy. The energy cutoff of 850 eV and the k-points sampling of $1\times 1\times 1$ is used in DFT calculations. The Fe-O-H interactions were modeled using a hydroxylated Fe_2O_3 (001) surface as described in ref. (*57*) which is repeated 4×4 on lateral dimension and kept vacuum space of 20 Å in our surface model. The model has surface hexagonal cell with six times a repetition of oxygen layers with three Oxygen atoms followed by two Fe layers with one Fe on each layer. The Fe terminated surface layers adsorbs OH and one-third of surface O adsorb one H

as described in ref. (*57*) The energy cutoff of 850 eV and the Γ-point sampling of Monkhorst-Pack set of k-points is used in spin-polarized DFT+U calculations (*15*) with U_{eff}= 4 eV for electrons in localized d-orbitals of Fe.

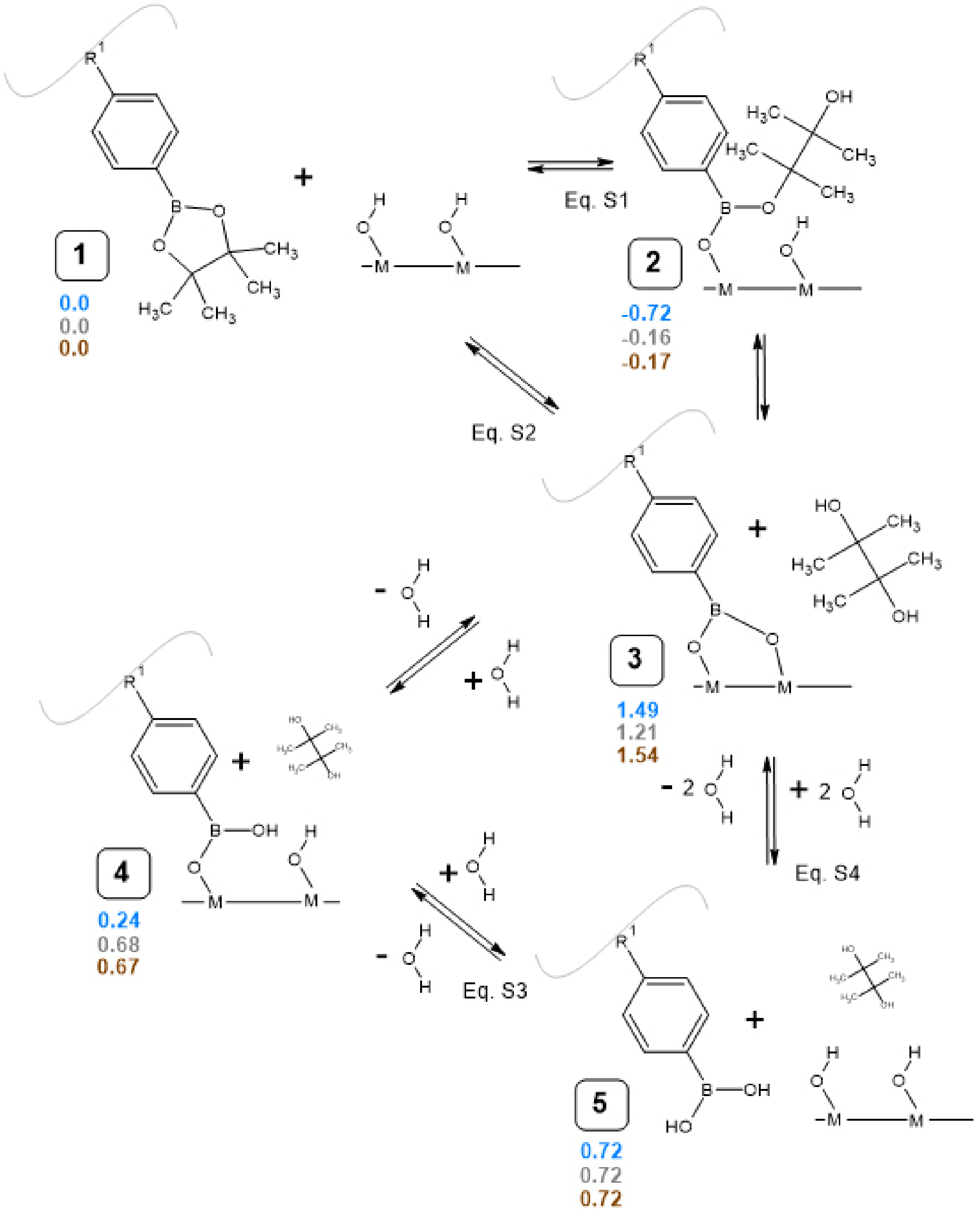

Fig. S16. **Mechanistic possibilities for Langmuir replacement reactions on a metal oxide when starting with the aryl-diboroxy ester used in this study**. State numbers are shown in squares. The R^1 group and the gray wavy line represent the location of the polymer backbone, and the horizontal line represents the surface of the metal oxide. The letter “M” represents lattice metal cations. For pictorial clarity, the gas phase pinacol molecule is not shown in States 4 and 5, and any liberated gas-phase water molecules are also not shown. Blue numbers represent the relative

electronic energies in eV for the silica surface, Grey numbers represent the relative electronic energies in eV for the alumina surface, Reddish-brown numbers represent the relative electronic energies in eV for the iron oxide surface: all electronic energies noted are relative to the initial state and are from **Table S4**, explained below.

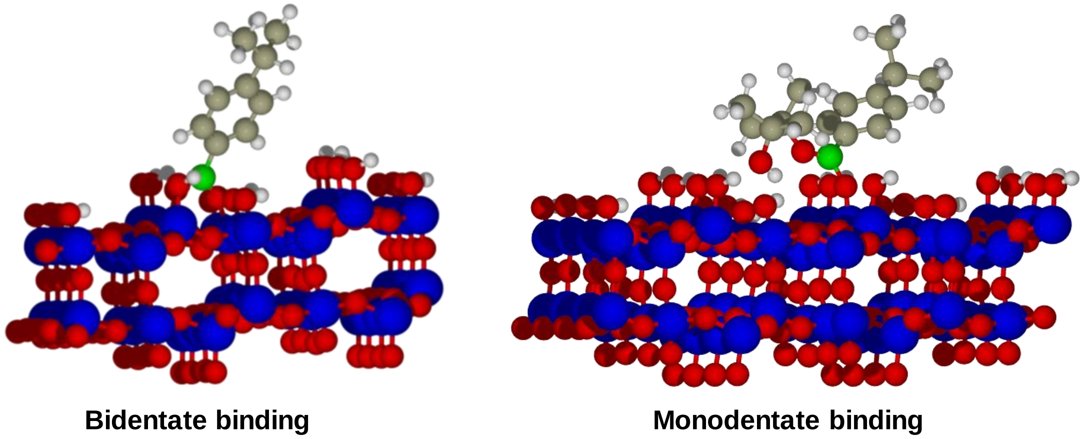

Fig. S17. Bi-dentate and mono-dentate binding of phenyl boronic ester on an SiO_2 surface.

DFT Calculated Energetics

We first optimized the pristine metal oxides, a silica (101) surface, a γ-alumina (0 0 1) surface, and a Fe_2O_3 (001) surface. We then introduced the molecule, substituting it for a terminating hydrogen (or two) followed by relaxation of the combined system (multiple geometric configurations were tried). The metal cations in the lattice (either Si or Al) are denoted "M" in the chemical equations below, and "MOx" refers to the metal oxide lattice upon which either OH groups or adsorbed molecules may be located. The oxygens that are part of OH groups or adsorbed molecules are considered external to the lattice for the purposes of the chemical equations, although these oxygens are bonded directly to the M cations. The reaction energy for replacement of M-OH groups and conversion to M-O-B-… products when starting from an initial state of a B-Aryl-Pin molecule ($E_{rep(B-Aryl-Pin)}$) or B-Aryl-OH molecule ($E_{rep(B-Aryl-OH)}$) can be calculated by

$$E_{rxn(B-Aryl-OH)} = E_{MOx-(B-Aryl-pinacoxy-O_1)} - (E_{MOx-OH} + E_{(B-Aryl-Pinacol)(g)})$$

(Eq. S1)

$$E_{rxn(B-Aryl-Pinacol)} = E_{MOx-(B-Aryl-O_2)} + E_{Pinacol\,(g)} - \left(E_{MOx-OH} + E_{(B-Aryl-Pinacol)(g)}\right)$$

(Eq. S2)

$$E_{rxn(B-Aryl-OH)} = E_{MOx-(B-Aryl-O_1)} + 1 * E_{H2O\,(g)} - (E_{MOx-OH} + E_{B-Aryl-OH(\mathrm{g})})$$

(Eq. S3)

$$E_{rxn(B-Aryl-OH)} = E_{MOx-(B-Aryl-O_2)} + 2 * E_{H2O\,(g)} - (E_{MOx-OH} + E_{B-Aryl-OH(\mathrm{g})})$$

(Eq. S4)

These reactions are shown in **Fig. S16** (note that the *equation* numbers should not be confused with the *state* numbers, as distinguished in **Table S4 and S6**. Equations S1 and S3 are for mono-

dentate adsorbates and Equations S2 and S4 are for bi-dentate adsorbates (note the subscripts in O_2 and O_1). The individual species energies, reaction energies, and state energies are shown in **Tables S4 – S6**, where for the reaction energies and the state energies a more negative is more energetically favored. The values in **Table S5** are not adsorption energies nor binding energies, but energies for the full replacement reaction to assess the favorability of the reaction. The term $\mathbf{E}_{\boldsymbol{MOx-OH}}$ is the energy of the OH saturated metal oxide slab before reaction. $\boldsymbol{E}_{\boldsymbol{Pinacol}\,(\boldsymbol{g})}$, and $\boldsymbol{E}_{(\boldsymbol{B-Aryl-Pinacol})(\boldsymbol{g})}$ are the total energy of a gas phase Pinacol molecule (including its 2 OH still intact) and the B-Aryl-Pinacol molecules, respectively. The term $\mathbf{E}_{\mathbf{MOx-(B-Aryl-O)}}$ refers to the total energy of substrate with adsorbed B-Aryl-O species after the loss of the pinacol functional group. The term $\mathbf{E}_{\mathbf{B-Aryl-OH}}$ refers to the total energy of the isolated B-Aryl-OH molecule. The energies of the individual species are included in **Table S4**. The reaction energies for S1 through S4 are provided in **Table S5**. While it is useful to have these reaction energies, it is more useful to consider the state energetics for the mechanistic network shown in **Fig. S16** on a common scale. This is done in **Table S6**. We cannot exclude the possibility that amorphous or otherwise curved oxide surfaces are capable of more strongly accommodating a bi-dentate configuration, but that was not seen in our calculations. In our calculations, we see that State 2, the mono-dentate state with pinacoxy moiety still bound is more stable than the bidentate state. We also see that humidity from the surroundings pushing the system to states 4 and 5 is not favored over any of the three oxide surfaces. Additionally, that even if humidity managed to push the system into states 4 and 5, a mono-dentate state would continue to remain favored.

Table S4. Molecular energy of different initial states used (gas molecules, surface states, adsorbed).

Species	Chemical Name	E
A	$H_2O(g)$	-14.22
B	$Pinacol(g)$	-118.79
C	$B - Aryl - OH\ (g)$	-152.10
D	$B - Aryl - Pinacol(g)$	-243.16
E	(Silica) $MOx - OH$	-3644.11
F	(Silica) $MOx - (B - Aryl - O_1)$	-3782.64
G	(Silica) $MOx - (B - Aryl - pinacoxy - O_1)$	-3888.15
H	(Silica) $MOx - (B - Aryl - O_2)$	-3767.15
I	(Alumina) $MOx - OH$	-4156.67
J	(Alumina) $MOx - (B - Aryl - O_1)$	-4294.59
K	(Alumina)$MOx - (B - Aryl - pinacoxy - O_1)$	-4399.99
L	(Alumina) $MOx - (B - Aryl - O_2)$	-4279.83
I	(Iron oxide) $MOx - OH$	-3706.85
J	(Iron oxide) $MOx - (B - Aryl - O_1)$	-3844.78
K	(Iron oxide)$MOx - (B - Aryl - pinacoxy - O_1)$	-3950.18
L	(Iron oxide) $MOx - (B - Aryl - O_2)$	-3829.68

Table S5. Reaction energies for the silica, alumina, and iron surfaces.

Surface	Reaction	Species Equation	E_{rxn} (eV)
Silica	Eq. S1	G-E-D	-0.716
Silica	Eq. S2	H+B-E-D	1.500
Silica	Eq. S3	F+A-E-C	-0.646
Silica	Eq. S4	H+2*A-E-C	0.617
Alumina	Eq. S1	K-I-D	-0.158
Alumina	Eq. S2	L+B-I-D	1.212
Alumina	Eq. S3	J+A-I-C	-0.041
Alumina	Eq. S4	L+2*A-I-C	0.495
Iron Oxide	Eq. S1	O-M-D	-0.172
Iron Oxide	Eq. S2	P+B-M-D	1.540
Iron Oxide	Eq. S3	N+A-M-C	-0.051
Iron Oxide	Eq. S4	P+2*A-M-C	0.823

Table S6. Comparison of state energies for the silica, alumina, and iron surfaces. ‡

Surface	State Number from **Fig. S16**	Species Equation	Absolute Energy (eV)	Relative Energy (eV)
Silica	1	E+D	-3887.27	0.00
Silica	2	G	-3888.15	-0.72
Silica	3	H+B	-3885.94	1.49
Silica	4	F+B-1*A	-3887.2	0.24
Silica	5	E+C+B-2*A	-3886.56	0.72
Alumina	1	I+D	-4399.83	0.00
Alumina	2	K	-4399.99	-0.16
Alumina	3	L+B	-4398.62	1.21
Alumina	4	J+B-1*A	-4399.15	0.68
Alumina	5	I+C+B-2*A	-4399.11	0.72
Iron Oxide	1	M+D	-3950.01	0.00
Iron Oxide	2	O	-3950.18	-0.17
Iron Oxide	3	P+B	-3948.54	1.54
Iron Oxide	4	N+B-1*A	-3949.33	0.67
Iron Oxide	5	M+C+B-2*A	-3949.29	0.72

‡States 4 and 5 have the energies subtracted of water molecules to account for the change in number of atoms in the electronic structure relative to State 1, as depicted in **Fig. S16**. Similarly, States 3 through 5 include the energy of a gas phase pinacoxyl molecule to ensure the absolute electronic structure energies are comparable.

Induced Charge Density Analysis

To identify the type of interaction between molecule and substrate, the induced charge density i.e. the difference in charge density (Δρ) between the combined system ($\rho_{SiO2-O-B-Aryl+H2O}$) and the isolated subsystem of $\rho_{B-Aryl-O1}$ (B-Aryl-OH without one H) molecule and substrate (ρ_{SiO2}) (SiO_2-OH without one OH at site where molecule adsorbs) is presented by calculating

$$\Delta\rho = \rho_{SiO2-(B-Aryl-O1)+H2O(g)} - \left(\rho_{\mathrm{SiO2}} + \rho_{B-Aryl-OH}\right). \quad (3)$$

The negative and positive Δρ correspond to the region where the electron density is lost and gain, respectively on forming combined system. This induced charge density analysis was only conducted for the most strongly bound state.

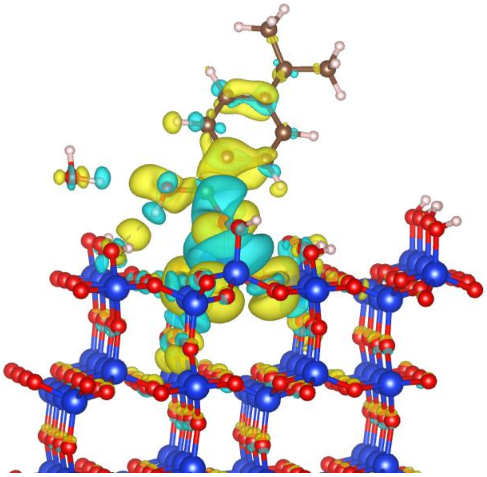

Fig. S18. Induced charge density of the mono-dentate B-Aryl-OH final state on the SiO_2-OH surface that shows charge gain (yellow) and depletion (cyan) regions on the system upon molecule adsorption. The iso-surface of the charge density difference is set at $9.7 \times 10^{-4}\ e/Å^3$.

The mono-dentate model of Species F, shown in **Fig. S17** is found to be energetically favored in which the molecule binds with energy > -0.72 eV reflecting a chemical bonding of the molecule on the substrate. The molecule aligns mostly vertical. The unreacted B-OH group forms a hydrogen bond to a nearby silanol Si-OH group on the surface, increasing the binding strength further. To show the type of bonding of the molecule on the surface, we also plot $\Delta\rho$ calculated from equation (3) in **Fig. S18**. The non-zero value of $\Delta\rho$ reflected from colors in the figure spreads over the region between atoms depicts the covalent bonding between the molecule and the surface atoms.
